# Development and characterization of a nanoemulsions-based semi-solid platform: Physicochemical properties, antioxidant and antimicrobial activities

**DOI:** 10.1016/j.ijpx.2026.100683

**Published:** 2026-10-24

**Authors:** Jacopo Forte, Linda Maurizi, Layla Musleh, Elvira Martino, Chiara Tammaro, Giovanna Poce, Isabel Schettini, Ana Isabel Fraguas Sanchez, Xcaret Flores Hernandez, Giusi Ianiro, Antimo Cutone, Barbara De Berardis, Federica Rinaldi, Maria Carafa, Catia Longhi

**Affiliations:** a Department of Basic Biotechnological Sciences, Intensivological and Perioperative Clinics, Catholic University of Sacred Heart, Largo Francesco Vito 1, Rome, 00168, Italy; b Department of Public Health and Infectious Diseases, Microbiology Section, Sapienza University of Rome, Piazzale Aldo Moro 5, Rome, 00185, Italy; c Department of Pediatric Surgery, San Camillo-Forlanini Hospital, Circonvallazione Gianicolense 87, Rome, 00152, Italy; d Department of Drug Chemistry and Technology, Sapienza University of Rome, Piazzale Aldo Moro 5, Rome, 00185, Italy; e CNR-IBPM, Piazzale Aldo Moro 5, Rome, 00185, Italy; f Department of Pharmaceutics and Food Technology, Complutense University of Madrid, Plaza Ramón y Cajal s/n, Madrid, 28040, Spain; g Department of Biosciences and Territory, University of Molise, C. da Fonte Lappone, Pesche, 86090, Italy; h National Centre Artificial Intelligence and Innovative Technologies for Health, Istituto Superiore di Sanità, Rome, 00161, Italy

**Keywords:** *Staphylococcus aureus*, Skin infections, Nanoemulsions, Lentisk oil, Levofloxacin

## Abstract

*Staphylococcus aureus* infection is a major contributor to chronic non-healing wounds, where persistent bacterial colonization and biofilm formation impair tissue repair and sustain prolonged inflammation. To counteract *S. aureus* skin colonization, levofloxacin (LVX)-loaded nanoemulsions (NEsL) were formulated using lentisk (*Pistacia lentiscus*) oil (LO) as the oily phase and incorporated into an advanced chitosan-based formulation (AF). The nanoemulsions combined the capacity to solubilize LVX, a lipophilic antibiotic used as a drug model, with the intrinsic antimicrobial activity of LO, employed as an active excipient. Both unloaded and drug-loaded NEsL were characterized in terms of particle size, biocompatibility, and antioxidant activity, demonstrating suitable physicochemical and biological properties. The AF was assessed for stability over time, film thickness, and adhesive performance; all formulations exhibited satisfactory bioadhesive properties and stability, supporting their potential for topical skin application.

*In vitro* and *ex vivo* permeation experiments on LVX-containing samples were conducted using a Franz diffusion cell system to evaluate drug permeation profiles and skin localization. Based on these findings, the most promising formulation, NEsL and AF loaded with NEsL (AF-NEsL), was selected and tested for its ability to counteract *S. aureus* skin colonization. Enhanced antimicrobial performance was observed exclusively when NEsL was incorporated into the AF matrix, compared to either NEsL or AF-NEs alone. Taken together, this bioadhesive nanoplatform based on bioactive nanocarriers may represent a promising approach for localized topical delivery, although its therapeutic efficacy requires further investigation.

## Highlights

• Innovative bioadhesive platform for persistent *Staphylococcus aureus* infections.
• Levofloxacin-loaded lentisk oil nanoemulsions for topical skin delivery.
• Chitosan-based adhesive matrix enhances nanoemulsion performance.
• Bioactive nanoemulsions exhibit suitable biocompatibility and antioxidant activity.
• Bioadhesive system enhances antimicrobial activity in infected skin models.

## Graphical abstract

**Figure:**
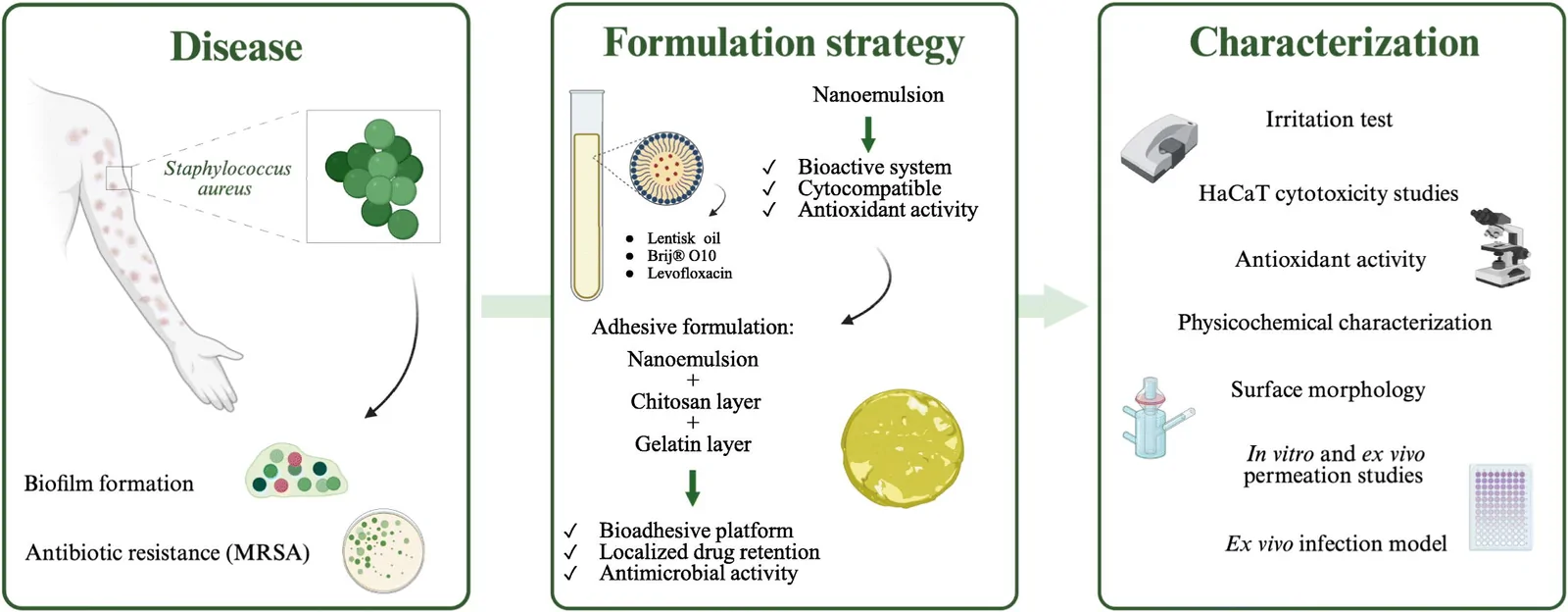

## 1 Introduction

*Staphylococcus aureus* is a versatile Gram-positive bacterium, which acts both as a commensal organism and an opportunistic pathogen, capable of causing a broad spectrum of human diseases, ranging from superficial skin and soft tissue infections to severe, life-threatening conditions such as pneumonia, endocarditis, osteomyelitis, septic arthritis, and bacteremia (Touaitia et al., 2025). Although it colonizes the anterior nares, skin, and mucosal surfaces of approximately 30% healthy individuals, *S. aureus* can exploit breaches in host barriers or immune defenses to establish invasive infections, highlighting its remarkable adaptability. The pathogenic success of *S. aureus* is driven by its numerous virulence factors along with its ability to form biofilms, which are structured microbial communities embedded within an extracellular polymeric matrix that contribute to the establishment of chronic infections. In particular, together with clinically relevant staphylococci such as *S. epidermidis*, *S. aureus* is recognized as a major factor in the long-term persistence and repeated occurrence of cutaneous infections, which contributes to treatment failure and exacerbates resistance to conventional therapies. The global rise of antibiotic resistance, including methicillin-resistant *S. aureus* (MRSA), has underscored the need for innovative therapeutic approaches (Touaitia et al., 2025; Lin et al., 2021).

Levofloxacin (LVX), a third-generation fluoroquinolone, is widely employed against Gram-positive skin pathogens, including *Staphylococcus* spp., due to its broad-spectrum bactericidal activity. However, systemic administration of LVX is associated with significant adverse effects and contributes to selective pressure driving resistance. The clinical utility of topical LVX is constrained by limited skin penetration and suboptimal activity against biofilm-embedded bacteria, highlighting the need for advanced delivery paradigms (Sarhan, 2024). Nanotechnology-based strategies have gained closer attention in recent years, offering potential solutions for enhanced antibiotic delivery and efficacy in skin application. Nanocarriers such as nanoemulsions, nanostructured lipid carriers, liposomes, niosomes and other lipid/surfactant-based systems can overcome limitations of conventional cutaneous treatments, improving drug solubility, stability, and skin penetration, while enabling sustained or targeted release at the site of infection (Martins et al., 2025; Teo et al., 2025).

A key characteristic of these nanocarriers is the ability to select specific components, such as oils, to enhance the overall formulation's therapeutic performance and increase the efficacy of the encapsulated active compound. Depending on its chemical composition, the oil component may exert antimicrobial, anti-inflammatory, or skin barrier-supporting effects, to improve both local bioavailability and therapeutic outcome (Kumar et al., 2024).

The strategy of the present work was to incorporate antibiotic-loaded nanoemulsions (NEsL) into a non-conventional advanced formulation (AF), combining the properties of the chitosan-based matrix with the intrinsic activity of oil-based nanoemulsions (NEs) due to the presence of lentisk (*Pistacia lentiscus*) oil (LO).

These nanoemulsions were designed to solubilize a lipophilic antibiotic such as LVX, used as a model drug, to evaluate the performance of NEs incorporated into the AF in enhancing the antimicrobial activity of the drug compared with the free form. The bioadhesive properties of AF could support formulation adherence and localized application at the skin surface (Teo et al., 2025; Almuqbil and Aldhubiab, 2025; Gonciarz et al., 2025a). AF could represent a biocompatible and biodegradable platform able to potentially support localized antibiotic delivery at cutaneous sites and maintain formulation integrity (Özkahraman et al., 2021). For this purpose, gelatin, a hydrolyzed form of collagen, able to produce a biocompatible and biodegradable gel, maintaining a moist microenvironment and supporting localized drug delivery, was chosen (Ahmady and Abu Samah, 2021). Although its intrinsic mechanical strength and adhesion are limited, gelatin's functional groups (amine, carboxyl, hydroxyl) facilitate hydrogen bonding with biological tissues and can be modulated to tune release kinetics. To potentially contribute to the adhesive properties of gelatin-based formulations, chitosan, a natural polysaccharide derived from deacetylated chitin, has been selected (Ebhodaghe, 2022). Moreover, its biodegradability and biocompatibility make it an ideal candidate for AF (Che et al., 2024).

Maurizi et al. (2024) previously demonstrated the antimicrobial and antibiofilm activity of LO-based NEs, particularly against some *Staphylococcus* spp. in both planktonic and sessile forms (Maurizi et al., 2024). While these findings established the therapeutic potential of LO-based nanoemulsions as antimicrobial nanocarriers, their application as localized delivery systems for cutaneous infections has not yet been explored. In particular, the limited residence time of conventional topical formulations on the skin may reduce the effectiveness of nanoemulsion-based treatments, highlighting the need for strategies able to prolong formulation retention at the site of infection. Based on these considerations, the novelty of the present work lies in the integration of previously developed LO-based nanoemulsions into AF, creating a multifunctional localized delivery platform. The NEs and NEsL, deeply investigated in previous work, were now analyzed in terms of biocompatibility, and antioxidant behavior. The AF was characterized in terms of stability over time, and qualitative adhesive performance. Unlike our previous study, which focused on the NEs characterization and their antimicrobial properties, the present work investigates whether the combination of LO-based nanoemulsions with AF. The ability of AF to modulate drug localization inside artificial membrane/skin localization was assessed through both in vitro and *ex vivo* models. Finally, the ability of the selected formulation to counteract *S. aureus* skin colonization was investigated.

## 2 Material and methods

### 2.1 Materials

Lentisk (*Pistacia lentiscus*) oil was kindly provided by Lentisk Soc. Coop. Valle del Cedrino “Costa degli Olivi” (Orosei, NU, Sardinia, Italy). Levofloxacin was supplied by Thermo Fisher Scientific Inc. (Waltham, MA, USA). Animal gelatin was obtained from Farmalabor (Canosa di Puglia, BT, Italy). Brij® O10 and HEPES salt (N-(2-hydroxyethyl)piperazine-N′-(2-ethanesulfonic acid) sodium salt), used for the preparation of HEPES buffer (pH 7.4), Glycerol (≥99.5%), low molecular weight chitosan, ethanol F.U., 2,2-diphenyl-1-picrylhydrazyl (DPPH), potassium ferricyanide, trichloroacetic acid, ammonium molybdate, iron chloride, dimethyl sulfoxide (DMSO) and 3-(4,5-dimethylthiazol-2-yl)-2,5-diphenyltetrazolium bromide (MTT) were purchased from Merck Life Science (Darmstadt, Germany). Glacial acetic acid and sulphuric acid were obtained from Carlo Erba Reagents (Milan, Italy).

### 2.2 Preparation of nanoemulsions

The optimal composition of NEs was determined using a ternary phase diagram constructed with LO, Brij® O10 and HEPES buffer (pH 7.4), as previously described in our last work. As reported by Maurizi et al., the selected formulation was processed by ultrasonication for 2 min at 50 °C using a tapered microtip probe operating at a frequency of 20 kHz and an amplitude of 20% (Vibracell-VCX 400, Sonics, Taunton, MA, USA) to obtain the desired nanoemulsions. LVX-loaded nanoemulsion, NEsL, was prepared following the same procedure adopted for the unloaded system, with the addition of LVX prior to the sonication step (Maurizi et al., 2024).

### 2.3 Cells

Normal human keratinocytes (HaCaT), purchased from Thermo Fisher Scientific (Thermo Fisher Scientific, Waltham, MA, USA), were maintained in a humidified atmosphere of 5% CO_2_ at 37 °C in Dulbecco's Modified Eagle Medium (DMEM, Corning Manassas VA, US) supplemented with 10% heat-inactivated fetal bovine serum (FBS, Euroclone, Italy) and 1% penicillin-streptomycin (Sigma-Aldrich, St. Louis, MO, USA) and 1% l-glutamine (Merck Life Science, Darmstadt, Germany).

### 2.4 NEs and NEsL characterization: irritation test, cytotoxicity, antioxidant activity and Dynamic Light Scattering

To establish suitable concentration ranges, the samples (NEs and NEsL) were formulated based on preliminary studies carried out prior to the final formulation. Following these results, the samples were diluted accordingly, and all experiments were conducted using the identified optimal concentrations.

#### 2.4.1 Irritation test

The potential irritancy of NEs and NEsL was evaluated in ovo using a modified hen's egg test–chorioallantoic membrane (HET-CAM) assay based on an endpoint assessment (European Commission, Joint Research Centre, 2026). Fertilized Leghorn chicken eggs (Granja Santa Isabel, Córdoba, Spain) were incubated in an Avimac C-100 incubator (Avimac, Basque Country, Spain) at 37 °C and 60% relative humidity with automatic rotation. On day 4 of incubation, the eggshell was carefully cleaned and gently drilled. The hole was sealed with adhesive tape to prevent desiccation. The eggs were then returned to the incubator under the same conditions but without rotation. On day 6, the opening was enlarged to expose the CAM, which was gently moistened with 1 mL of physiological saline solution (0.9% NaCl). A silicone ring (1 cm in diameter) was placed on the CAM surface to delimit the treatment area. Subsequently, 300 μL of each sample was applied within the ring: HEPES buffer (negative control), 0.1 M NaOH (positive control), and the tested nanoemulsions (NEs and NEsL). Each condition was evaluated using six eggs (*n* = 6). After a fixed exposure time of 3 min, the treated area was rinsed with physiological saline solution, and the CAM was further observed for an additional 2 min to detect the presence of vascular damage (hemorrhage, lysis, and coagulation). Images of each egg were acquired immediately before and 5 min after each treatment using a DM9 Max microscope (Tomlov, LA, USA). Vascular damage was scored independently in each egg using a semi-quantitative scale (0 = no reaction, 1 = weak reaction, 2 = moderate reaction, 3 = strong reaction). For each condition, scores were summed across the six eggs for treatment, and the highest total score was considered the final irritation score (S-score). Treatments with S-score ≤ 12 were considered non- or slightly irritating, those with scores of 13–15 irritating, and those with scores ≥16 severely irritating.

#### 2.4.2 Cytotoxicity studies on keratinocytes

The cell metabolism of HaCaT cells was measured by MTT (3-(4,5-dimethylthiazol-2-yl)-2,5-diphenyltetrazolium bromide) assay. Briefly, 96-well plates were seeded with 3 × 10^5^ /mL HaCaT cells, and, after 48 h of incubation, monolayers were treated with different NEs and NEsL concentrations ranging from 2.5 μg/mL to 0.16 μg/mL. After 24 h, the medium was removed, the cells were washed with phosphate-buffered saline (PBS) and, subsequently, incubated with 0.5 mg/mL MTT for an additional 4 h. Finally, the medium was removed, the cells were washed with PBS three times, and then 100 μL of DMSO was added to dissolve the formazan crystals. Optical density (OD) at 570 nm was determined with a microplate reader (PerkinElmer, Hopkinton, MA), and the cell viability for each concentration was estimated through comparison with untreated cells. DMSO at 10% *v*/v was used as a positive control. The experiment was performed in three independent biological replicates (*n* = 3).

#### 2.4.3 Antioxidant activity

##### 2.4.3.1 DPPH free radical scavenging activity assay

To assess and compare the antioxidant capacity of NEs and NEsL, the DPPH free radical scavenging assay was performed with modifications (Baj et al., 2018). Both NEs and NEsL were prepared at five different concentrations: 25, 50, 75, 100 and 125 μg/mL. 1 mL of each nanoparticle suspension was mixed with 1 mL of a methanolic DPPH solution at 4% (*v*/v). The resulting mixtures were then incubated in the dark at room temperature for 12 h to allow interaction between the nanoparticles and DPPH radicals, after incubation the mixtures were centrifuged at 10.000 rpm for 4 min to remove insoluble components. The decrease in DPPH radical levels, reflecting antioxidant activity, was determined by measuring the absorbance at 517 nm using a UV–Vis spectrophotometer of the supernatants. The percentage of radical scavenging activity was calculated according to the following equation:

$$\text{Scavenging activity}\;\left(\%\right)=\left[\left(A\_\text{control}-A\_\text{sample}\right)/A\_\text{control}\right]\times 100$$

where A_control corresponds to the absorbance of the DPPH solution without nanoparticles, and A_sample refers to the absorbance in the presence of NEs or NEsL.

##### 2.4.3.2 Reducing power assay

To evaluate the antioxidant potential of NEs and NEsL, a ferric-reducing power assay was performed (Akhtar et al., 2022). NEs and NEsL were prepared at five different concentrations (25, 50, 75, 100 and 125 μg/mL). For each concentration, 1 mL of nanoparticle solution was mixed with 1 mL of 1% potassium ferricyanide and 1 mL of phosphate buffer (0.2 M, pH 6.6). The mixtures were then incubated at 50 °C for 20 min to allow the reduction of ferric ions. After incubation, 0.5 mL of 10% (*w*/*v*) trichloroacetic acid was added. Subsequently, 1 mL of this solution was mixed with 1 mL of deionized water and 0.125 mL of 0.1% ferric chloride solution. The absorbance was measured at 700 nm using a UV–Vis spectrophotometer, where increased absorbance indicates higher reducing power and, consequently, greater antioxidant activity. The assay was performed in three independent experiments (*n* = 3).

##### 2.4.3.3 Assessment of total antioxidant capacity

To determine the total antioxidant capacity (TAC) of NEs and NEsL, a series of nanoparticle concentrations (25, 50, 75, 100 and 125 μg/mL) was prepared (Akhtar et al., 2022). For each concentration, the nanoparticle solution was combined with 1 mL of a reaction mixture consisting of 0.6 M sulphuric acid, 0.1 M sodium phosphate buffer, and 0.28 M ammonium molybdate. The mixtures were then incubated at 95 °C for 90 min to allow the formation of the phosphomolybdenum complex, which indicates antioxidant activity. Following incubation, the samples were allowed to cool to room temperature. The absorbance was measured at 695 nm using a UV–Vis spectrophotometer. The total antioxidant capacity was subsequently calculated and expressed as ascorbic acid equivalents by comparing the absorbance of the samples with that of a blank (without nanoparticles) and a standard ascorbic acid solution. All five concentrations (25–125 μg/mL) were initially evaluated; however, only concentrations yielding absorbance values within the reliable range of the ascorbic acid calibration curve were considered suitable for quantitative determination of TAC. The assay was performed in three independent experiments (*n* = 3).

##### 2.4.3.4 Antioxidant activity on keratinocytes

HaCat cells were seeded at a density of 1 × 10^5^ cells per well in 24-well plates and allowed to reach a confluence of approximately 90–95%. Cells were pre-treated for 3 h with NEs or NEsL at final concentrations of 1.25, 0.63, 0.31, and 0.16 μg/mL, previously determined to be non-cytotoxic, and then challenged with 0.5 mM H_2_O_2_. After 24 h of treatment, cells were collected, and cell counts were performed manually using a THOMA chamber (Merck Life Science, Milan, Italy). The experiment was performed in three independent biological replicates (*n* = 3).

#### 2.4.4 Dynamic Light Scattering and ζ-potential measurements

Dynamic Light Scattering (DLS) was employed to determine the hydrodynamic diameter, size distribution, and ζ-potential of samples, which were analyzed at the optimal concentration identified and used throughout the experiments (Brognaro et al., 2019). Measurements were carried out using a Malvern Nano ZS90 instrument (Malvern Instruments, Worcestershire, UK), equipped with a 5 mW HeNe laser operating at 632.8 nm and configured at a fixed scattering angle of 90°. All samples were analyzed at room temperature. The intensity autocorrelation functions were processed using the cumulant and Contin algorithms to obtain the mean hydrodynamic diameter (DH) and the polydispersity index (PDI), with particle size values reported as intensity-weighted averages (Koppel, 1972). The PDI was used to evaluate the homogeneity of the colloidal systems, with values below 0.3 indicating a narrow and uniform size distribution, a key requirement for reproducible behavior and reliable performance in drug delivery applications.

The ζ-potential was determined from the electrophoretic mobility of the vesicles measured by Laser Doppler Anemometry. The electrophoretic mobility (μ) was converted into ζ-potential according to the Smoluchowski equation (ζ = μη/ε), where η and ε represent the viscosity and permittivity of the dispersing medium, respectively (Gopmandal and Duval, 2022). ζ-potential values with an absolute magnitude above ±30 mV were considered indicative of good colloidal stability, due to the electrostatic repulsion between particles, and were also taken as an important parameter influencing nanoparticle–cell interactions and internalization behavior (D'Intino et al., 2025). The experiment was performed in three independent replicates (*n* = 3).

### 2.5 Preparation of empty and loaded AF

Preliminary optimization studies were carried out to define the final formulation parameters. Different qualitative–quantitative compositions were prepared by varying the concentration of components and process parameters, as described in Supplementary Information. The final formulation was chosen through an iterative process guided by predefined experimental criteria, following a stepwise and systematic screening process. Based on these preliminary investigations, the optimized formulation was defined. Specifically, a gelatin layer was prepared by dissolving 2% (*w*/w) gelatin and 1% (*w*/w) glycerol in an aqueous medium under magnetic stirring at 60 °C for 1 h until complete solubilization was achieved. Subsequently, 2 g of the resulting solution was poured into silicone molds and dried at 40 °C for 5 h, resulting in the formation of a uniform gelatin layer. Concurrently, the chitosan layer was prepared by dissolving 2% (*w*/w) chitosan in an aqueous solution containing 1% (*w*/w) glacial acetic acid. The mixture was magnetically stirred for 2 h at room temperature to ensure complete polymer dissolution. The resulting solution was then centrifuged at 8000 rpm for 10 min. NEs and NEsL, previously prepared as described in Section 2.2, were incorporated into the chitosan solution at a 1:1 (*w*/w) ratio and mixed under magnetic stirring for 30 min to obtain a homogeneous dispersion. An aliquot of 2.5 g of the resulting formulation was then uniformly spread onto the preformed gelatin layer and dried in a ventilated oven. The obtained AF was finally stored at room temperature in a glass desiccator containing silica gel until further use. The formulations containing NEs and NEsL were identified as AF-NEs and AF-NEsL, respectively. The AF formulation corresponds to the base formulation without nanoemulsions, while AF-LVX contains free levofloxacin dispersed in the AF matrix.

The composition of empty and loaded AF is reported in Table 1.

**Table 1:** Composition of the final advanced formulations.

Sample	Gelatin (% *w*/w)	Glycerol (% w/w)	Chitosan (% w/w)	Glacial acetic acid (% w/w)	NEs (% w/w)	NEsL (% w/w)	LVX (% w/w)
**AF**	0.89	0.44	0.56	0.28	–	–	–
**AF-LVX**	0.89	0.44	0.56	0.28	–	–	0.03
**AF-NEs**	0.89	0.44	0.56	0.28	27.78	–	–
**AF-NEsL**	0.89	0.44	0.56	0.28	–	27.78	–

Abbreviations: AF, advanced formulation without nanoemulsions; AF-LVX, advanced formulation containing free levofloxacin; AF-NEs, advanced formulation containing empty nanoemulsions; AF-NEsL, advanced formulation containing nanoemulsions loaded with levofloxacin.

### 2.6 Measurement of the AF thickness

The thickness of advanced formulations (AF, AF-LVX, AF-NEs and AF-NEsL) was measured using a digital thickness gauge, Scienceware Digi-Max (Sigma-Aldrich, Switzerland), equipped with a slide caliper with an accuracy of ±0.1 mm. Measurements were performed at a central point on each sample at room temperature (Gautam et al., 2023). The experiment was performed in three independent replicates (*n* = 3).

### 2.7 Adhesion studies

To evaluate the potential skin-retention properties of the AF, an inclined plane test was performed using a glass substrate model. To better mimic the physiological hydration conditions of the skin surface, the glass slide was lightly moistened with a minimal amount of distilled water and gently blotted to remove excess liquid prior to formulation application. This qualitative method is commonly employed to assess the resistance of semisolid topical formulations to gravitational flow and their ability to potentially remain in situ after application (Al-Barghouthy et al., 2025).

Briefly, a fixed amount of formulation (AF, AF-LVX, AF-NEs, AF-NEsL) was carefully applied onto a clean glass slide, which was then positioned at a 90° inclination. At predetermined time intervals (5, 15, 30, and 60 min), formulation retention was qualitatively evaluated by visual inspection, based on its ability to remain adhered to the inclined substrate. All experiments were conducted at room temperature (Chang et al., 2013).

### 2.8 Surface morphology of AF

Surface morphology of AF was investigated by Scanning Electron Microscopy (SEM) analysis (FE-SEM Quanta Inspect, FEI Company, Eindhoven, The Netherlands). Samples were frozen in liquid nitrogen and freeze-dried for 24 h (Jaiswal et al., 2016). Portions of the freeze-dried samples were mounted on SEM stubs, coated with a thin Au film by sputtering and analyzed by SEM. Image editing was performed by Adobe Photoshop CS4 software (Adobe Systems, San Jose, CA, USA).

### 2.9 *In vitro* and *ex vivo* permeation studies

#### 2.9.1 Franz cells diffusion *in vitro* study: Strat-M

In vitro permeation studies were performed on free LVX, NEsL, AF-LVX, and AF-NEsL, using vertical Franz diffusion cells with an effective diffusion area of 2.5 cm^2^ to investigate the permeation behavior of the LVX present in the formulations. The diffusion system consisted of a donor compartment (1 mL capacity), in which the sample was placed, and a receptor compartment (5 mL capacity) filled with the selected receptor medium. A Strat-M® synthetic membrane (Millipore, Sigma-Aldrich) was placed between the two compartments to mimic human skin permeability, as this polymeric membrane is widely used as a skin surrogate for in vitro permeation studies (Kichou et al., 2023) (Fig. 1). Franz diffusion cells were maintained at 32 °C to simulate physiological skin temperature using a thermostatically controlled water bath (Haake SPP/SC100, Thermo Scientific), while continuous stirring of the receptor phase was ensured by magnetic agitation (Alhasso et al., 2023). At predetermined time intervals (every hour), aliquots were withdrawn from the receptor compartment for UV–visible spectrophotometric analysis (Lambda 25, PerkinElmer, Waltham, MA, USA) and subsequently returned to the receptor medium to maintain a constant volume. The permeation experiment was carried out for up to 5 h. The percentage of LVX permeated through the Strat-M® membrane (F%) at each time point was calculated from the amount of drug recovered in the acceptor compartment, according to the following equation:

$$F\%=\left(\mathrm{Fₓ}/\mathrm{Fₜ}\right)\times 100$$

where Fₓ represents the amount of active compound released at time x, and Fₜ is the total amount of active compound initially loaded into the formulation.

**Fig. 1:**
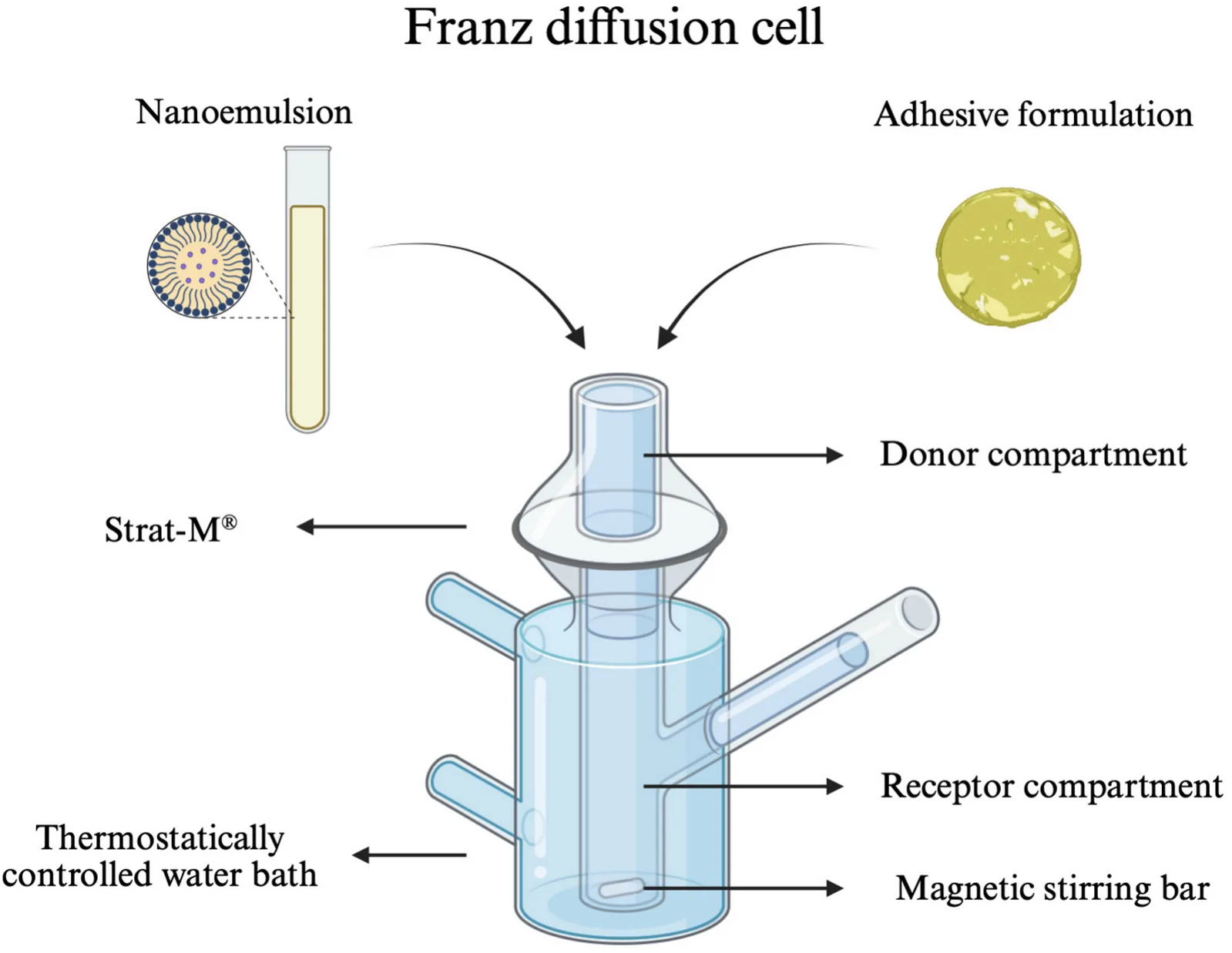
Schematic illustration of the Franz diffusion cell apparatus used for *in vitro* permeation studies across Strat-M® synthetic membrane. Created with BioRender.com.

At the end of the experiment, the Strat-M® membrane was collected and immersed in ethanol to extract the fraction of active compound retained within the membrane, which was subsequently quantified by UV–visible spectroscopy. Furthermore, the LVX remaining in the donor compartment was recovered and quantified to complete the mass balance evaluation. The experiment was performed in three independent replicates (*n* = 3).

#### 2.9.2 Franz cells diffusion *ex vivo* study: porcine ear

##### 2.9.2.1 Preparation of porcine ear skin

Porcine ear skin was obtained from a local Italian supplier and stored at −20 °C for a period not exceeding three months. Prior to use, the skin samples were thawed, cleaned, and carefully shaved to remove hair. Circular skin sections with a diameter of 1 cm were prepared using a sterile scalpel. The thickness of each skin section was measured using a digital thickness gauge (Scienceware Digi-Max, Sigma-Aldrich, Switzerland; accuracy ±0.1 mm). These sections were subsequently used for permeation and infection experiments.

##### 2.9.2.2 *Ex vivo* permeation studies using Franz diffusion cells

Skin discs with a diameter of 0.5 cm were prepared and then used for the film permeation study. The experiment was carried out as described in Section 2.9.1, with the difference that, instead of the Strat-M® membrane, porcine ear skin was placed between the donor and receptor compartments of the Franz diffusion cells (Fig. 2).

**Fig. 2:**
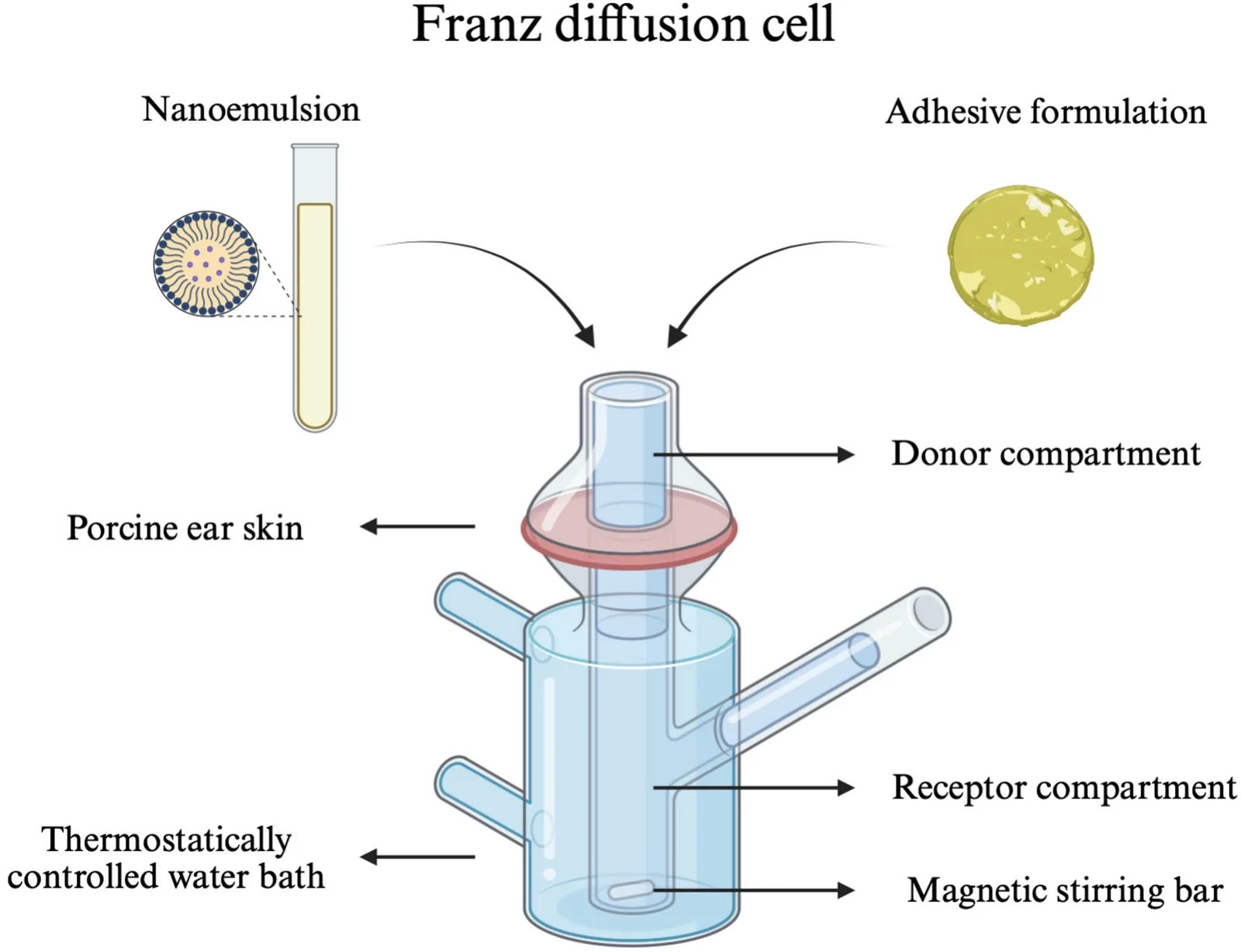
Schematic illustration of the Franz diffusion cell apparatus used for *ex vivo* permeation studies across porcine ear skin. Created with BioRender.com.

NEsL and AF-NEsL were applied onto the stratum corneum and the percentage of LVX permeated through the skin (F%) at each time point was calculated using the following equation:

$$F\%=\left(\mathrm{Fₓ}/\mathrm{Fₜ}\right)\times 100$$

where Fₓ represents the amount of LVX detected in the receptor compartment at time x, and Fₜ is the total amount of LVX initially loaded into the NEs. Additionally, for control purposes, the amount of LVX was measured in the donor compartment and in the porcine ear skin. The experiment was performed in three independent replicates (*n* = 3).

### 2.10 Stability studies of AF

The stability of AF, AF-NEs, and AF-NEsL was assessed by monitoring changes in both physical and visual properties. Physical stability was evaluated by tracking weight variations to detect potential evaporation of the aqueous component, with measurements performed at days 1, 30, 60, and 90 under two storage conditions (25 °C and 4 °C). Simultaneously, visual stability was monitored over the same period, under the same conditions to detect any observable changes (Serra et al., 2025). The experiment was performed in three independent replicates (*n* = 3).

### 2.11 Bacterial strains

The bacterial strain used in this study was the reference strain *Staphylococcus aureus* ATCC 6538P, obtained from the American Type Culture Collection (ATCC, Manassas, VA, USA). The bacterial strain was cultured overnight in Brain Heart Infusion (BHI) broth at 37 °C and stored in 15% glycerol-BHI at −80 °C. The minimum inhibitory concentration (MIC) of NEs was determined against this strain using the broth microdilution method and performed in triplicate as previously described (Maurizi et al., 2024).

### 2.12 *Ex vivo* infection model

To evaluate the antimicrobial activity of the different preparations, *ex vivo* studies were performed using porcine ear skin, prepared as described above, and infected with *S. aureus* ATCC 6538P. Skin discs were placed in sterile 24-well plates and infected with exponentially growing bacterial cultures at a concentration of 1 × 10^6^ colony-forming units (CFU)/mL. Skin samples were incubated at 37 °C for 1 h to allow bacterial adhesion. After incubation, the samples were exposed to a sub-inhibitory concentration of NEs and NEsL (0.05 μg/μL), or of AF-NEs and AF-NEsL (0.3 μg/μL), and further incubated for 3 h at 37 °C. Following treatment, each skin disc was transferred into a sterile tube containing 5 mL of PBS. Samples were vigorously vortexed for 5 min to detach adherent bacteria. The resulting suspensions were serially diluted, plated on Tryptic Soy Agar (TSA), and incubated at 37 °C for 24 h. The experiment was performed in three independent biological replicates (*n* = 2).

### 2.13 Statistical analysis

For antimicrobial studies, cytotoxic and antioxidant activity on keratinocytes, Franz cells diffusion assays, and *ex vivo* infection model, statistical analysis was conducted using one-way ANOVA followed by Tukey's post hoc pairwise tests (GraphPad Prism, Version 5.0). A *p*-value of less than 0.05 (* *p* < 0.05) was considered statistically significant.

For the reducing power and TAC assays, data were analyzed using two-way ANOVA followed by Šídák's multiple-comparisons test, as specified in the corresponding figure legend. A *p*-value of less than 0.05 (* *p* < 0.05) was considered statistically significant.

## 3 Results and discussion

The use of an oil-based nanocarrier, combined with antimicrobial agents, represents a promising strategy to enhance the efficacy of topical treatments for persistent skin infections.

In our previous study, Maurizi et al. (2024), a comprehensive physicochemical characterization of the nanoemulsions was carried out, including the determination of hydrodynamic diameter, ζ-potential, morphology (TEM analyses), drug entrapment efficiency, stability, as well as in vitro release and permeation profiles. Furthermore, the antimicrobial and antibiofilm properties of the formulations were investigated against both reference and clinical *Staphylococcus* spp. isolates. Overall, NEs were characterized by a hydrodynamic diameter of 165.7 nm and a monodispersive distribution; these results were confirmed by visual inspection by TEM analyses. Moreover, the E.E. % was evaluated and the amount of LVX entrapped in the nanocarriers was 100%. The developed nanoemulsions exhibited excellent long-term stability, maintaining hydrodynamic diameter and ζ-potential unchanged for up to 90 days under both refrigerated and room-temperature storage conditions likely due to suitable ζ-potential values (≈ −30 mV). In vitro release studies were carried out, showing complete levofloxacin release (≈100% within 3 h). This result confirmed the ability of the NEsL to release the loaded drug and supported their suitability for further studies focused on skin application and drug permeation. Compared with the free antibiotic, the antibiotic-loaded nanoemulsions demonstrated superior antimicrobial efficacy, particularly against sessile *Staphylococcus* spp.

Levofloxacin-loaded nanoemulsions significantly enhanced the eradication of pre-formed *Staphylococcus* biofilms, particularly in clinical *S. aureus* isolates, achieving eradication rates of up to 71%, outperforming the free antibiotic (Maurizi et al., 2024).

NEsL demonstrated complete LVX encapsulation, indicating efficient drug solubilization capability and suitable physicochemical features for the proposed aim. Furthermore, NEsL retained MIC values relative to free LVX, effectively delivering the antibiotic in the growth medium while preserving its activity, and exhibited significant antibiofilm activity against *S. aureus* clinical strains at low sub-inhibitory concentrations (Maurizi et al., 2024). The aim of this study was to develop an advanced chitosan-based semi-solid formulation (AF) capable of incorporating previously prepared and characterized NEsL, with the goal of enhancing their effectiveness in the treatment of skin disorders. Initially, irritation studies were conducted to rule out potential adverse effects of the nanoemulsions on the skin, along with cytotoxicity assays on keratinocytes to assess their safety profile.

### 3.1 Irritation test

Building on the previously developed and physicochemically characterized NEsL formulation, whose antimicrobial activity has already been demonstrated, the present study specifically investigated its suitability for topical application. Given its potential use in the treatment of skin disorders, particular attention was devoted to the assessment of skin tolerability and irritation potential, as essential prerequisites for safe dermatological use. The tolerability of the developed NEs was evaluated in ovo using the HET-CAM assay, a widely accepted method for evaluating irritation and local toxicity of dermatological formulations through the observation of vascular responses in the CAM (Fraguas-Sánchez et al., 2022).

As shown in Fig. 3, NEsL exhibited no signs of toxicity, as no evidence of hemorrhage, vascular lysis, or coagulation was observed in the treated eggs. Accordingly, an S-score of 0 was obtained. Similarly, eggs treated with NEs and HEPES buffers (negative control) did not exhibit any signs of irritation (S-score = 0). In contrast, eggs treated with 0.1 M NaOH (positive control) exhibited clear vascular damage, including hemorrhage, lysis, and coagulation, resulting in an S-score of 16, indicating that it is a highly irritating compound. This effect is consistent with previous studies reporting the strong irritant effect of NaOH (da Silva et al., 2026). Overall, the absence of detectable toxicity in the HET-CAM assay supports the potential suitability of NEsL for topical administration.

**Fig. 3:**
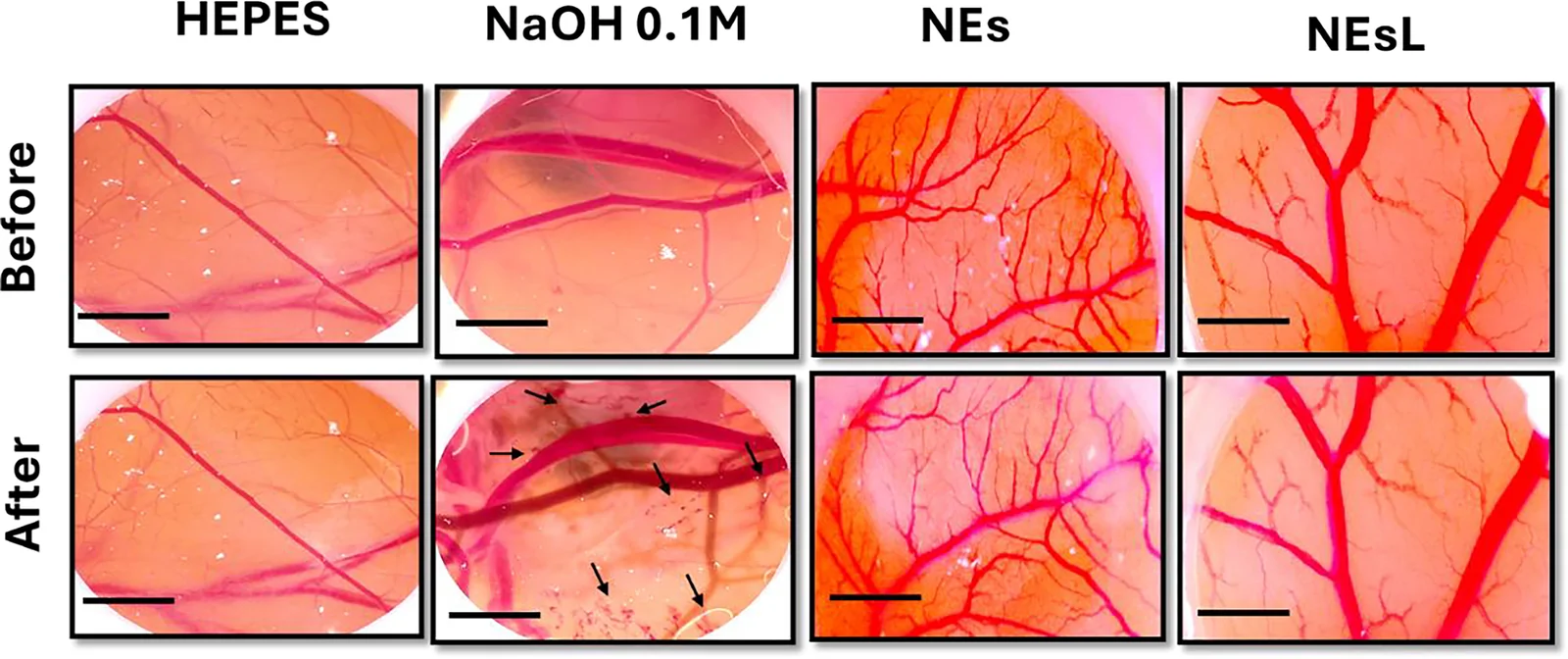
Evaluation of the irritation potential of nanoemulsions using the HET-CAM assay. Representative images of the chorioallantoic membrane before and after treatment with HEPES buffer (negative control), 0.1 M NaOH (positive control), NEs and NEsL. Black arrows indicate areas of vascular damage. Scale bar: 0.25 cm.

### 3.2 Cytotoxicity studies on keratinocytes

Keratinocytes constitute approximately 95% of epidermal cells and represent the most important cell type in human epidermal tissues (Park et al., 2021). Therefore, the human immortalized keratinocyte cell line HaCaT was selected to evaluate the skin toxicity of preparations. Briefly, the cell viability of NEs and NEsL was evaluated after 24 h of contact with cell monolayers using the MTT assay. The nanoemulsions were tested at concentrations ranging from 2.5 μg/mL to 0.16 μg/mL. As shown in Fig. 4, NEs and NEsL exhibited cytotoxic effects only at the highest tested concentrations. A slight difference in cell viability between the LVX-loaded and unloaded formulations was detected. These findings align with those produced by other authors that demonstrated that the incorporation of LVX into the nanoemulsion significantly reduced its toxicity (Razdan et al., 2022). Furthermore, according to other studies that investigated the potential of nanoformulations constituted by essential oils, free LO exhibited cytotoxic effects at all tested concentrations (data not shown), suggesting that the encapsulation system may modulate the release of LO and limit its direct contact with the cells (Fahmy et al., 2023; Özakar et al., 2024).

**Fig. 4:**
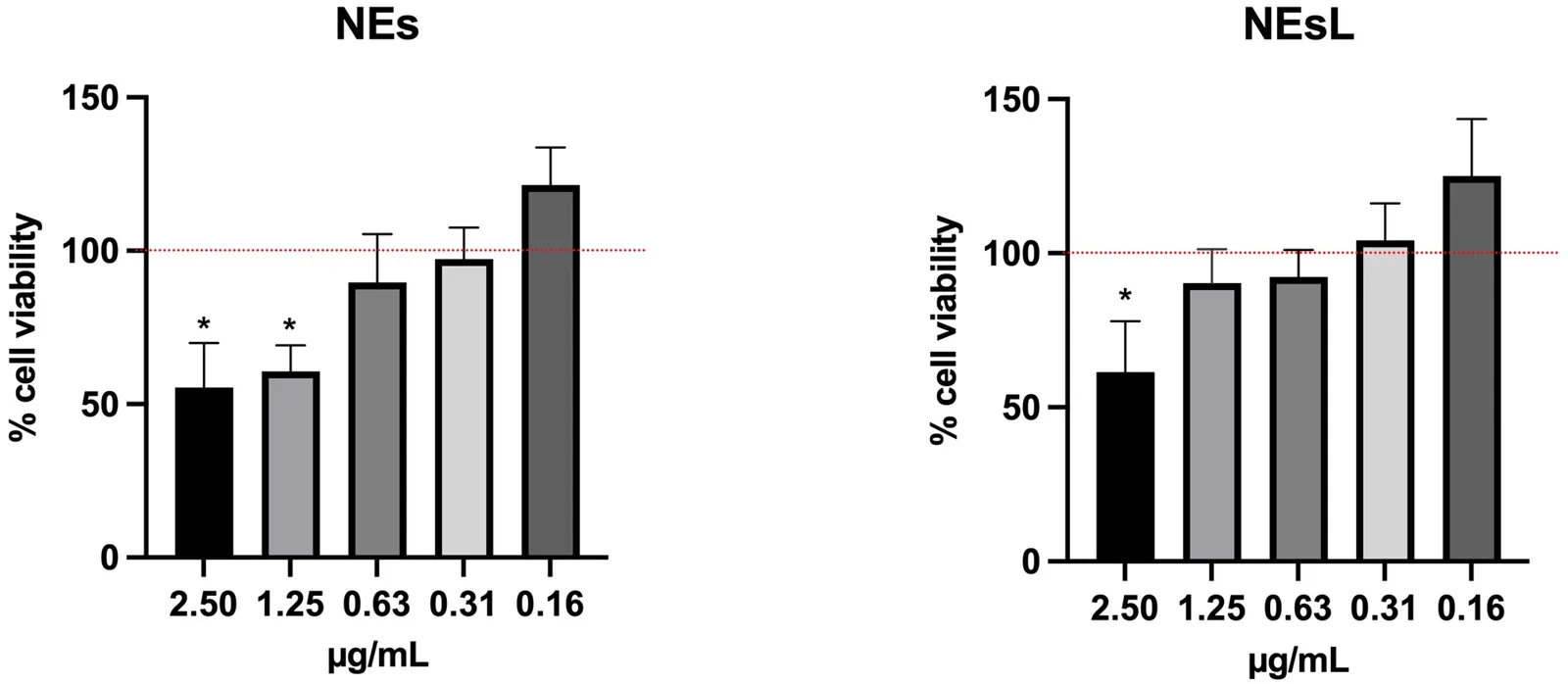
Percentage of HaCaT cell viability after 24 h exposure to different concentrations of NEs and NEsL, normalized to the untreated control (100%). Error bars: standard error of the mean. Asterisks (*) indicate a statistically significant difference compared to the control group (*: p < 0.05) (one-way ANOVA with post-hoc Tukey test).

### 3.3 Antioxidant activity

#### 3.3.1 DPPH free radical scavenging activity assay

The radical scavenging activity of the nanoformulations was preliminarily investigated using the DPPH assay, based on the ability of antioxidants to neutralize DPPH free radicals through hydrogen- or electron-donating mechanisms. Both NEs and NEsL nanoemulsions were evaluated over a concentration range of 25–125 μg/mL. Initial measurements were strongly affected by optical interference, likely due to the intrinsic turbidity and light-scattering properties of the nanoemulsion systems. To address this issue, a centrifugation step was introduced prior to analysis, which improved the reliability of the measurements. The results showed a decrease in absorbance values compared to the control (A = 1.280), particularly at lower concentrations, suggesting radical scavenging activity for both NEs and NEsL. The highest radical scavenging activity was observed at 25 μg/mL, reaching 66.4% for NEsL and 54% for NEs, with NEsL showing a higher scavenging value than NEs under these conditions. However, an atypical concentration-dependent trend was observed. While lower concentrations resulted in a clear reduction of DPPH absorbance, increasing the concentration led to progressively higher absorbance values, in some cases exceeding that of the control. This non-linear behavior suggests that residual optical interference and matrix effects remain relevant at higher concentrations. These effects are likely associated with increased particle density, aggregation phenomena, and light scattering within the nanoemulsion system in the methanol-containing medium. NEsL exhibited lower absorbance values than NEs up to 100 μg/mL, which may suggest a relatively higher scavenging capacity of the loaded formulation, particularly at lower concentrations. The reference antioxidant, ascorbic acid, showed absorbance values consistently lower than the control, supporting the performance of the assay. Overall, the DPPH results should be interpreted as preliminary qualitative evidence of radical-scavenging activity, since the non-linear concentration-dependent response and residual optical interference prevent a robust quantitative comparison between the two formulations.

#### 3.3.2 Reducing power assay

The reducing power assay evaluates the ability of the formulations to reduce Fe^3+^ to Fe^2+^, reflecting their electron-donating capacity and antioxidant potential. The data revealed that both NEsL and NEs exhibited concentration-dependent changes in reducing power, although a non-linear behavior was observed when comparing the two formulations (Fig. 5, Panel A). At low concentration (25 μg/mL), NEs showed a higher absorbance (0.2130 ± 0.0111) compared to NEsL (0.1081 ± 0.0098; *p* < 0.0001), indicating a higher reducing capacity in the absence of LVX. This difference between the two formulations was also observed at 50 μg/mL, although it became progressively less pronounced with increasing concentration. At the intermediate concentration of 75 μg/mL, the two systems exhibited comparable values (0.3318 ± 0.0048 for NEs and 0.3391 ± 0.0134 for NEsL). Conversely, at higher concentrations, the trend appeared to shift, with NEsL reaching a higher absorbance than NEs at 125 μg/mL (0.4679 ± 0.0068 vs 0.4309 ± 0.0058, respectively; *p* < 0.001), suggesting a possible contribution of LVX to the reducing activity at the highest concentration tested. Overall, the convergence of the two profiles at 75 μg/mL followed by their inversion at the highest concentration suggests that the differences in reducing power between unloaded and LVX-loaded nanoemulsions vary depending on concentration.

**Fig. 5:**
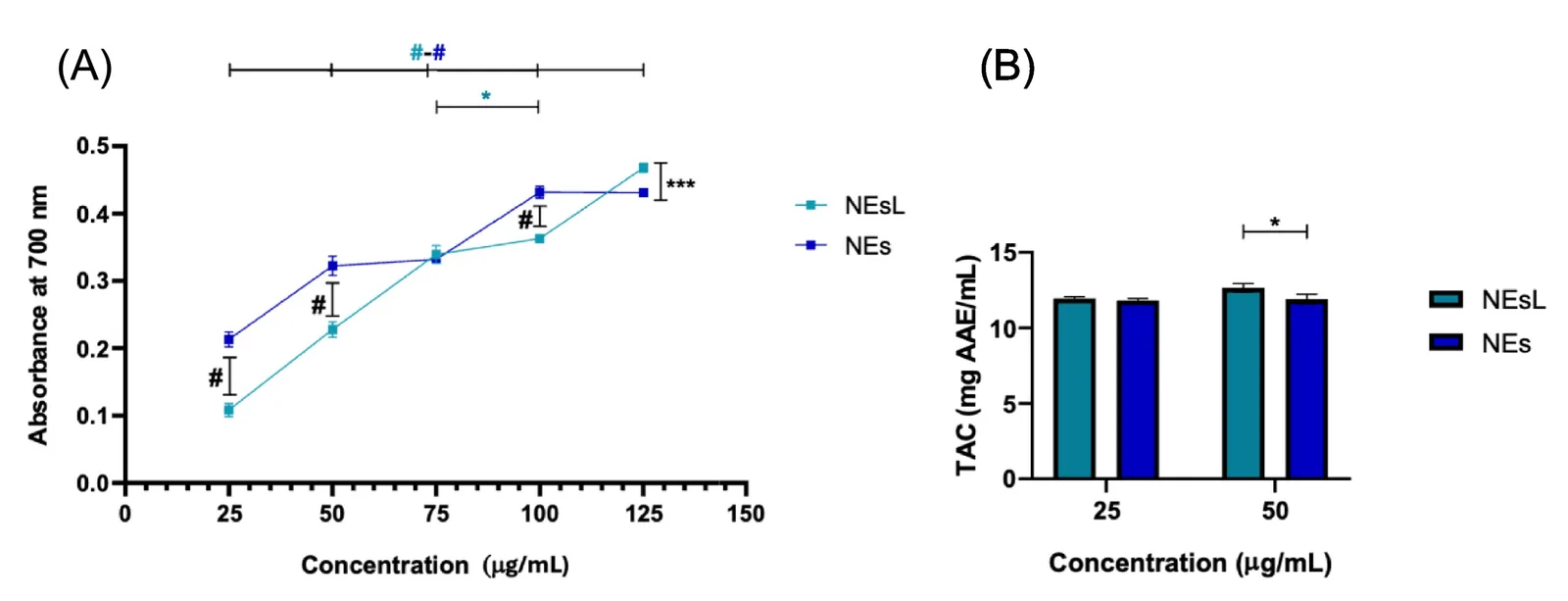
Panel A: Reducing power assay of unloaded nanoemulsions (NEs) and levofloxacin-loaded nanoemulsions (NEsL) evaluated over the concentration range 25–125 μg/mL. Panel B: Total antioxidant capacity (TAC) of unloaded nanoemulsions (NEs) and levofloxacin-loaded nanoemulsions (NEsL) at 25 and 50 μg/mL. In Panel A, horizontal comparison bars indicate pairwise comparisons among concentrations within the same formulation, with symbol color identifying the corresponding formulation, whereas vertical comparison bars indicate comparisons between NEs and NEsL at the same concentration. Statistical significance is indicated as follows: *: p < 0.05, ***: p < 0.001, and #: p < 0.0001 (one-way ANOVA with post-hoc Tukey test).

#### 3.3.3 Assessment of total antioxidant capacity

TAC was evaluated by comparing the absorbance values of the samples with those of a control solution consisting of HEPES buffer treated with all assay reagents (A = 0.2166) and an ascorbic acid reference solution. A decrease in absorbance relative to the control was interpreted as an indication of potential antioxidant activity under the investigated conditions. A calibration curve was constructed using ascorbic acid standard solutions, showing the relationship between concentration and absorbance. Among the five concentrations initially evaluated, only 25 and 50 μg/mL yielded absorbance values within the reliable range of the calibration curve and were therefore considered suitable for quantitative analysis. At higher concentrations (75–125 μg/mL), the absorbance values fell outside the reliable calibration range, likely due to matrix-related interference such as light scattering or turbidity associated with the nanoemulsion system (Komaiko and McClements, 2016). Therefore, these concentrations were excluded from quantitative analysis. The calibration equation was subsequently used to estimate the total antioxidant capacity of NEs and NEsL at 25 and 50 μg/mL in terms of ascorbic acid equivalents (AAE).

In particular, NEsL exhibited AAE values of 11.94 and 12.67 μg/mL at 25 and 50 μg/mL, respectively, while NEs showed values of 11.83 and 11.92 μg/mL at the same concentrations (Fig. 5, Panel B). No significant difference between NEsL and NEs was observed at 25 μg/mL, whereas at 50 μg/mL NEsL exhibited a significantly higher TAC than NEs (*p* < 0.05). This difference, in line with the trend observed in the DPPH assay, may reflect a contribution of LVX to the overall antioxidant capacity of the loaded formulation. However, the antioxidant response that could be reliably quantified was restricted to a limited concentration range. Overall, these findings provide preliminary *in vitro* evidence of antioxidant activity for both nanoformulations.

#### 3.3.4 Antioxidant activity on keratinocytes

Taking into account the cytotoxicity assessment, the antioxidant and cytoprotective effects of the formulations were evaluated in HaCaT cells exposed to hydrogen peroxide (H₂O₂, 0.5 mM). The nanoemulsions were tested at concentrations of 1.25, 0.63, 0.31, and 0.16 μg/mL (Fig. 6). Treatment with H₂O₂ alone reduced cell viability by approximately 50%, confirming the induction of oxidative stress conditions.

**Fig. 6:**
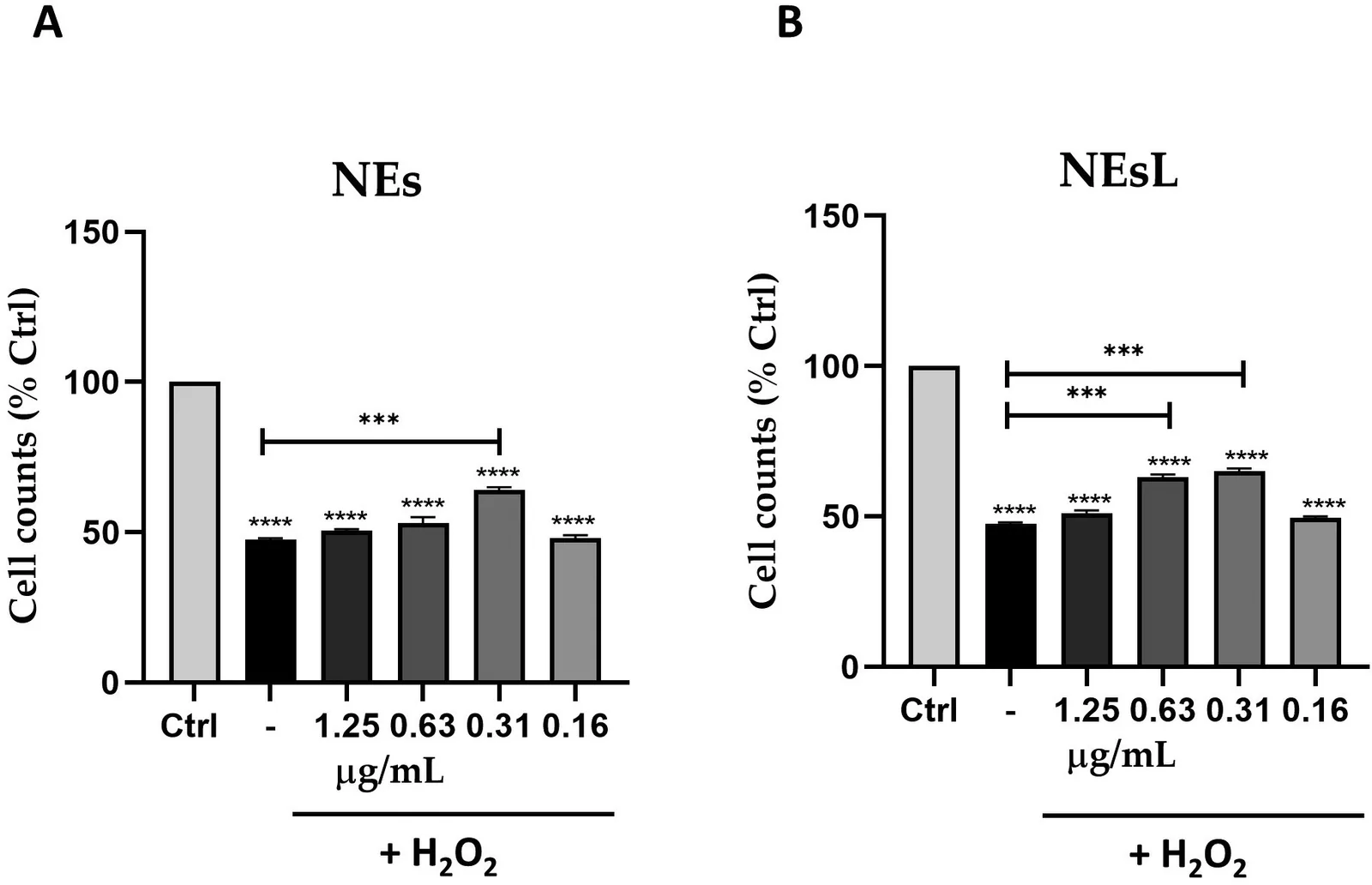
Cell proliferation assay to evaluate the antioxidant and cytoprotective effect of NEs (A) and NEsL (B) against H₂O₂-induced oxidative stress in HaCat cells. Cells were pre-incubated for 3 h with different concentrations of NEs and NEsL and then challenged with 0.5 mM H₂O₂. Error bars: standard error of the mean. Asterisks (*) indicate a statistically significant difference compared to the control group; horizontal bars with asterisks indicate a significant difference between specific groups (***: p < 0.001; ****: p < 0.0001) (one-way ANOVA with post-hoc Tukey test).

NEs did not show significant protective effects at the higher concentrations tested (1.25 and 0.63 μg/mL), whereas a significant recovery in cell viability was observed at 0.31 μg/mL. NEsL exhibited a similar trend, with a significant increase in cell viability at intermediate concentrations (0.63 and 0.31 μg/mL). At the lowest concentration tested (0.16 μg/mL), no protective effect was observed for either formulation.

Overall, the results suggest a concentration-dependent cytoprotective effect for both formulations within a limited concentration range. These findings are in agreement with the cytotoxicity data, where both NEs and NEsL showed minimal toxicity at lower concentrations, supporting the identification of a concentration window in which the formulations are well tolerated and able to partially counteract oxidative stress without inducing relevant cytotoxic effects.

### 3.4 Dynamic Light Scattering and ζ-potential measurements

After identifying the concentration at which the nanoemulsions were non-cytotoxic, the physicochemical properties of NEs and NEsL were re-evaluated. Specifically, particle size, polydispersity index (PDI), and ζ-potential were measured to confirm that the nanocarriers retained their characteristic features at the selected biocompatible concentration. As shown in Table 2, both formulations displayed particle size, PDI, and ζ-potential values comparable to those of NEs and NEsL previously characterized (Maurizi et al., 2024).

**Table 2:** Physicochemical properties of Nanoemulsions.

Sample	Hydrodynamic diameter ± SD (nm)	PDI ± SD	ζ-potential ± SD (mV)
**NEs**	160.55 ± 2.09	0.13 ± 0.03	−27.81 ± 1.23
**NEsL**	168.37 ± 0.93	0.17 ± 0.05	−30.21 ± 1.56

### 3.5 Preparation of AF and loaded AF

The AF was developed to support the localized application of NEsL on the affected skin area; for this purpose, a chitosan-based semi-solid formulation was prepared.

Chitosan, a linear cationic polysaccharide widely used in dermal formulations, is able to potentially adhere to mucosal and skin surfaces thanks to electrostatic interactions between its positively charged amino groups and the negatively charged components of the stratum corneum and extracellular matrix. These interactions may support formulation adherence at the application site, potentially contributing to localized topical delivery. Such characteristics have been extensively reported in the literature, where chitosan-based gels have demonstrated prolonged residence on the skin surface and reduced formulation runoff compared to non-adhesive vehicles (Hamedi et al., 2022). Several optimization experiments, documented in the Supplementary Information and shown in Fig. S1, were performed to achieve an optimized AF with respect to thickness, consistency, appearance, and reproducibility. Empty and loaded AF were obtained following the optimized preparation method described in Section 2.5. All samples, AF, AF-LVX, AF-NEs, and AF-NEsL, exhibited similar thickness values, as reported in Table 3, suitable for the proposed skin application (Chellathurai et al., 2021; Prajapati et al., 2011; Cherukuri et al., 2017).

**Table 3:** Thickness values of the advanced formulation.

Sample	AF	AF-LVX	AF-NEs	AF-NEsL
**Thickness ± SD (mm)**	0.29 ± 0.01	0.30 ± 0.03	0.35 ± 0.02	0.32 ± 0.03

As shown in Fig. 7, empty AF and AF containing free LVX were clear and transparent, whereas formulations containing nanoemulsions (both NEs and NEsL) showed a yellowish coloration, due to the characteristic color of the lentisk oil used in the nanoemulsion formulations.

**Fig. 7:**
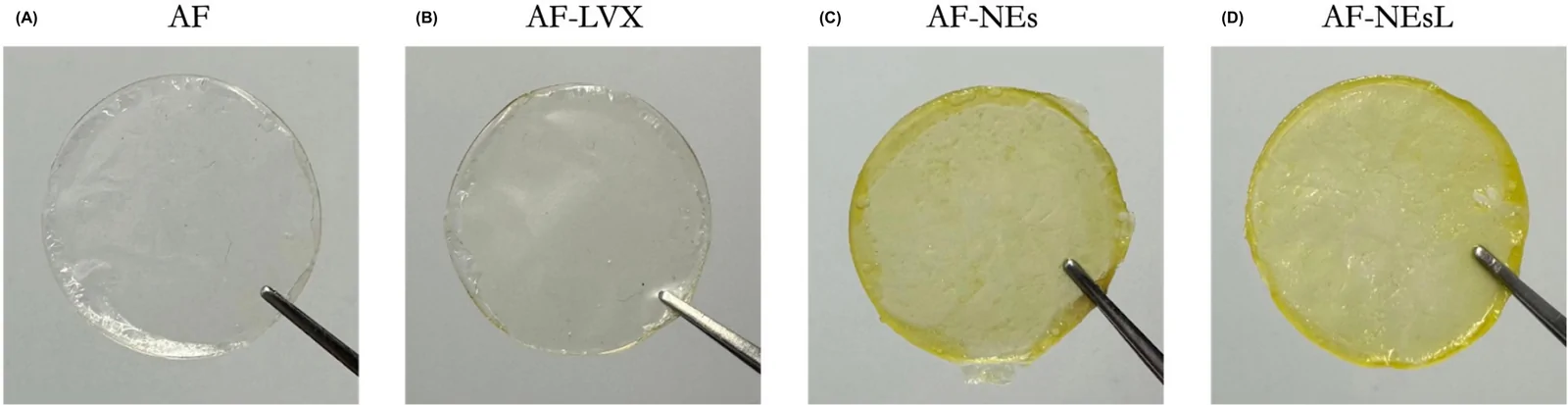
Representative images of the prepared advanced formulations (AF, AF-LVX, AF-NEs and AF-NEsL).

### 3.6 Adhesion studies

In order to evaluate the potential adhesive properties of AF, probably due to the presence of chitosan, a qualitative sliding test was performed using a vertical glass substrate model, following previously reported methods, as a preliminary assessment of formulation adherence. The glass slide was positioned at a 90° angle, and all samples (AF, AF-LVX, AF-NEs, AF-NEsL) were applied onto its surface. The tendency of the formulations to slide off the substrate was assessed by visual inspection over time. The experiments were conducted at room temperature and observed for 60 min. All samples remained on the inclined slide during the observation period, indicating resistance to gravitational displacement under the selected experimental conditions. As none of the samples detached from or slid off the substrate within the observation period, a quantitative detachment or sliding time could not be determined using this assay; the test only established that all formulations remained on the substrate for at least 60 min under the selected conditions. In particular, as shown in Fig. S2, formulations containing NEs appeared to remain closer to their original position, whereas the other samples showed minor displacement.

### 3.7 SEM morphological analysis

AF formulation exhibited a homogeneous and compact surface crisscrossed by long strands with some agglomerates of polymeric particles (Fig. 8A). The surface of AF-LVX formulation showed similar morphological characteristics, with agglomerates of polymeric particles between the strands that run through the film (Fig. 8B). The surface morphology of the AF-NEs formulation was very different; in fact, it was rich in NEs flocculates that organized into very dense and compact three-dimensional structures (Fig. 8C). In the AF-NEsL formulation, the NEsL merged to form short, narrow strands that intertwined and branched across the film surface, increasing the roughness of the AF (Fig. 8D).

**Fig. 8:**
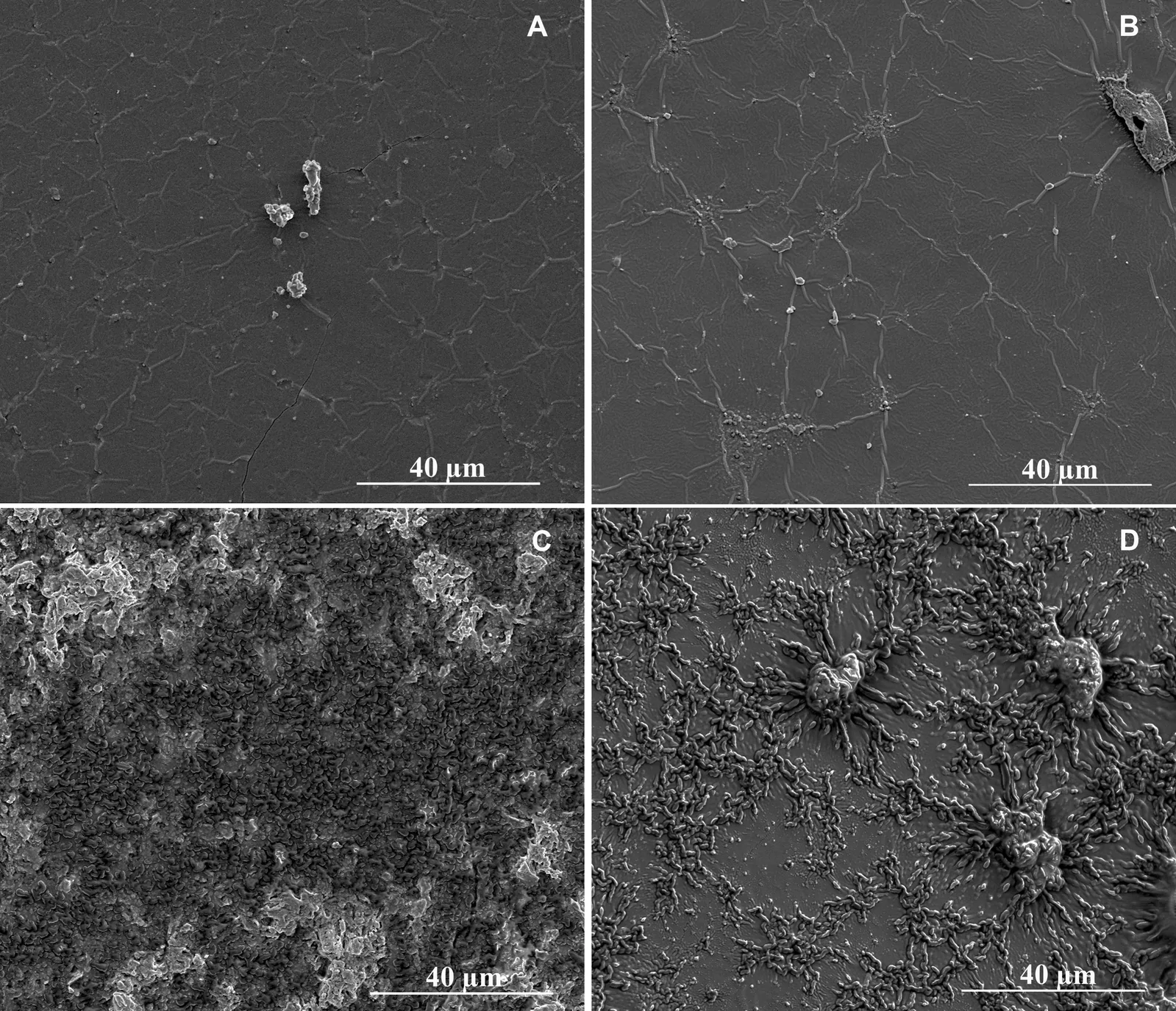
SEM image of surface of lyophilized formulations: A) AF; B) AF-LVX; C) AF-NEs; D) AF-NEsL.

### 3.8 *In vitro* and *ex vivo* permeation studies

The permeation experiments were performed to investigate the interaction of LVX with the Strat-M®/skin tissue and its permeation through the tissue.

#### 3.8.1 Franz cells diffusion *in vitro* studies: Strat-M

The NEsL were placed in the donor compartment of Franz diffusion cells under the previously described experimental conditions. The permeation and retention of LVX on the membrane were monitored via UV spectroscopy. The study was specifically designed to evaluate the drug capability to permeate a Strat-M® synthetic membrane, which mimics the barrier properties of human skin (Uchida et al., 2015), and in particular to quantify the fraction of drug that crosses the membrane, remains in the acceptor compartment, or is retained within the membrane.

In particular, from Fig. 9, it is possible to observe that the permeation across the Strat-M® membrane was limited to approximately 10% for all formulations, while a higher fraction of free LVX, 30%, was detected in the acceptor compartment. According to Franz diffusion cell methodology, the presence of this amount of drug in the receptor compartment is considered evidence of permeation across the membrane, supporting a potential transdermal delivery behavior (Jonsdottir et al., 2025).

**Fig. 9:**
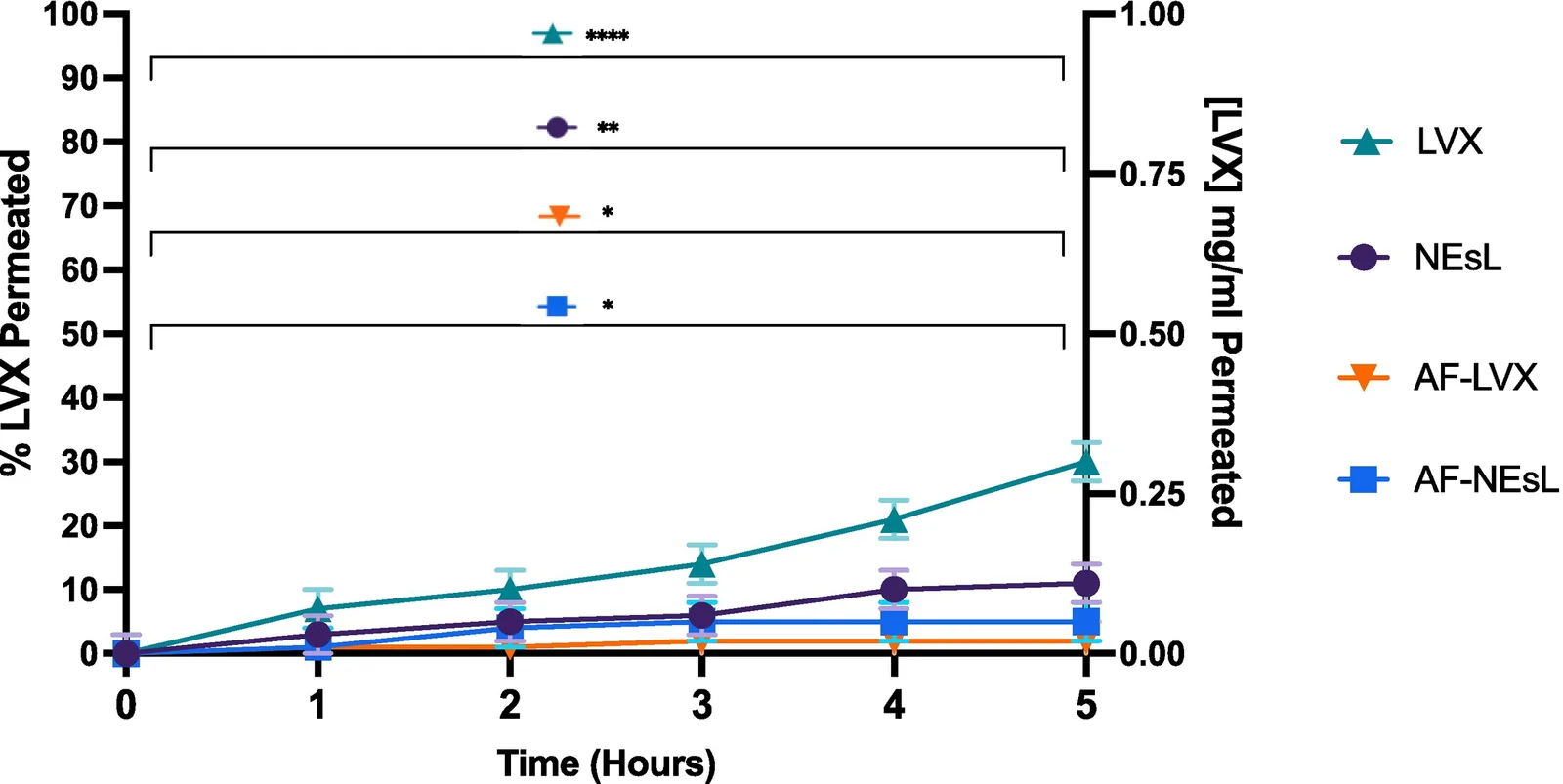
*In vitro* permeation studies of LVX through Strat-M® in a Franz cell system. The cumulative amount of LVX permeated into the acceptor compartment is plotted as a function of time. Error bars: standard error of the mean. Horizontal bars with asterisks (*) indicate a significant difference between specific groups (*: p < 0.05; **: p < 0.01; ****: p < 0.0001) (one-way ANOVA with post-hoc Tukey test).

For AF containing free LVX, the majority of the drug remained in the donor compartment (Table 4), with no interaction of LVX with strat-M indicating that the formulation was unable to effectively interact with the membrane, thus potentially limiting its potential for topical skin delivery (Arce et al., 2020).

**Table 4:** Percentage recovery of LVX in the donor and acceptor compartments and percentage retention in the Strat-M® membrane following in vitro permeation studies. Values are expressed as a percentage of the initial dose in NEsL.

Sample	LVX detected in Donor Compartment (%)	LVX detected in Acceptor Compartment (%)	LVX retained in Strat-M® membrane (%)
**LVX**	9	30	61
**NEsL**	14	11	75
**AF-NEsL**	21	5	74
**AF-LVX**	97	2	1

The best-performing formulations appeared to be NEsL and AF-NEsL, as in both cases only a small fraction of levofloxacin was detected in the acceptor or donor compartments, as shown in Fig. 9 and Table 4. The majority of the drug, higher than 70%, was retained within the Strat-M membrane, indicating strong interaction with the barrier and limited permeation. This observation suggests that the NEs are capable of efficiently bringing LVX into contact with the Strat-M membrane, potentially enhancing local drug delivery (Kaur et al., 2018).

#### 3.8.2 Franz cells diffusion *ex vivo* studies: porcine ear

##### 3.8.2.1 Preparation of porcine ear skin

Pig skin has been shown to possess histological and physiological characteristics similar to human skin, making it a suitable model for different research. Both pig and human skin feature a keratinized epidermis and dermis, with a comparable organization of cells in the stratum corneum, which serves as a barrier regulating permeability. Due to these similarities, it is frequently used in drug permeation studies. The thickness of the epidermis and stratum corneum in pig skin closely matches that of human skin, a factor critical for accurately assessing drug diffusion (Barbero and Frasch, 2009). In our experiments the thickness was measured and found to be about 0.8 ± 0.1 mm.

##### 3.8.2.2 *Ex vivo* permeation studies using Franz diffusion cells

To confirm the data obtained from the Franz diffusion cell permeation study, *ex vivo* experiments were performed using porcine ear skin as a substitute for the synthetic M® membrane. The experiments were performed with the best formulations, NEsL and AF-NEsL, that showed promising results with Strat-M® assays.

From fig. 10 and Table 5 it is possible to observe that the percentage of LVX permeated did not exceed 15% after 5 h for both samples. This slightly higher value compared to the Strat-M® experiments was expected due to the intrinsic differences between the synthetic membrane and skin; however, a similar behavior was observed, with approximately the same fraction of levofloxacin retained by the skin (70%) as was observed with the Strat-M® membrane, indicating that the majority of the drug remains associated with the tissue. These findings support the suitability of Strat-M® as a predictive model for skin permeation studies and suggest preferential localization of LVX within the skin, potentially favoring local topical delivery rather than transdermal permeation (Yu et al., 2014).

**Fig. 10:**
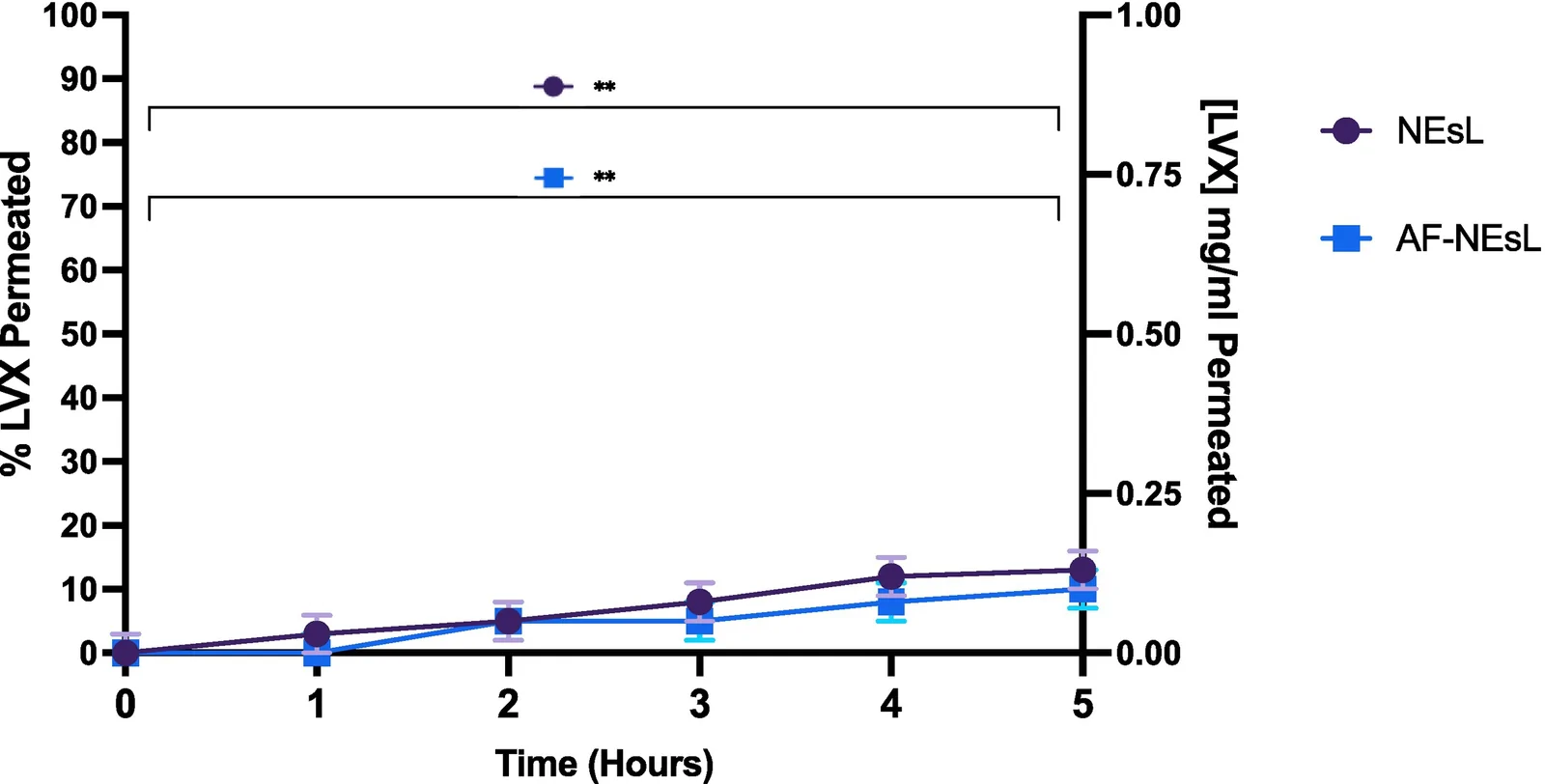
*Ex vivo* permeation studies of LVX through porcine ear skin in a Franz cell system. The cumulative amount of LVX permeated into the acceptor compartment is plotted as a function of time. Error bars: standard error of the mean. Horizontal bars with asterisks (*) indicate a significant difference between specific groups (**: p < 0.01) (one-way ANOVA with post-hoc Tukey test).

**Table 5:** Percentage recovery of LVX in the donor and acceptor compartments and percentage retention in the porcine ear skin following ex vivo permeation studies. Values are expressed as a percentage of the initial dose in NEsL.

Sample	LVX detected in Donor Compartment (%)	LVX detected in Acceptor Compartment (%)	LVX retained in porcine ear skin (%)
**NEsL**	16	13	71
**AF-NEsL**	18	10	72

Both the NEsL and AF-NEsL exhibited similar efficiency; for this reason, both formulations were further investigated for their local antimicrobial efficacy.

### 3.9 Stability studies of AF

The present stability assessment focused primarily on the macroscopic and physical characteristics of empty and loaded AF, as the evaluation of NEs stability within such a complex matrix is technically challenging.

In particular, as described in the Materials and Methods section, both visual stability assessments and gravimetric analyses were conducted for 90 days under two storage conditions: refrigerated temperature (4 °C) and room temperature (25 °C).

Visual inspections were carried out to detect any macroscopic alterations, including phase separation, surface irregularities, cracking, brittleness, deformation, or structural collapse of AF. Such evaluations are commonly employed as preliminary indicators of physical instability in chitosan-based semi-solid formulations, either empty or loaded with NEs, as macroscopic changes often reflect underlying structural rearrangements, polymer relaxation phenomena, or incompatibilities between components (Hamedani et al., 2025).

In parallel, gravimetric stability studies were performed to determine potential weight variations over time. This analysis was aimed at identifying any loss of volatile components, such as residual solvents or water, which could evaporate during storage and consequently affect film mechanical properties, flexibility, or drug release performance. Monitoring weight constancy of the formulation is particularly relevant for chitosan-based systems, since moisture content is essential for preserving the mobility of chitosan polymer chains and the integrity of their structure (Sutharsan et al., 2022).

Over the 90-day observation period, for all samples at both temperatures, no visible macroscopic alterations were detected under both storage conditions (Fig. 11).

**Fig. 11:**
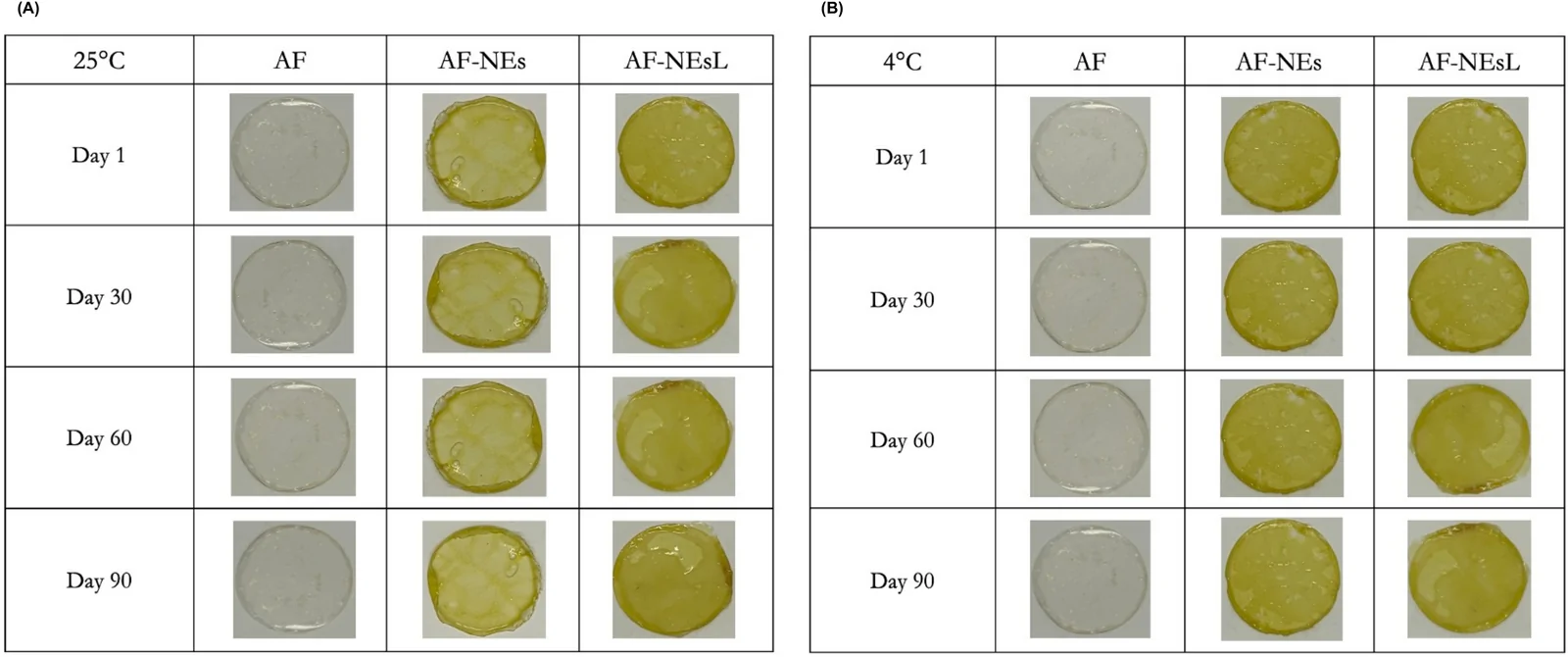
Storage stability studies conducted to AF, AF-NEs and AF-NEsL.

In particular, for all samples, the AF maintained its structural integrity (Fig. 12). In fact, visual inspection was performed to monitor potential changes in the appearance of the formulations during storage, including changes in color (as oxidation phenomena), phase separation, precipitation, or other visible alterations. No appreciable changes in appearance were observed over the study period, suggesting that the formulations maintained their macroscopic physical integrity. Furthermore, no appreciable weight loss was observed, suggesting the absence of significant evaporation during storage. Probably, the chitosan matrix provided adequate structural stabilization of the system, and the incorporation of nanoemulsions did not appear to destabilize the system (Thomas et al., 2017).

**Fig. 12:**
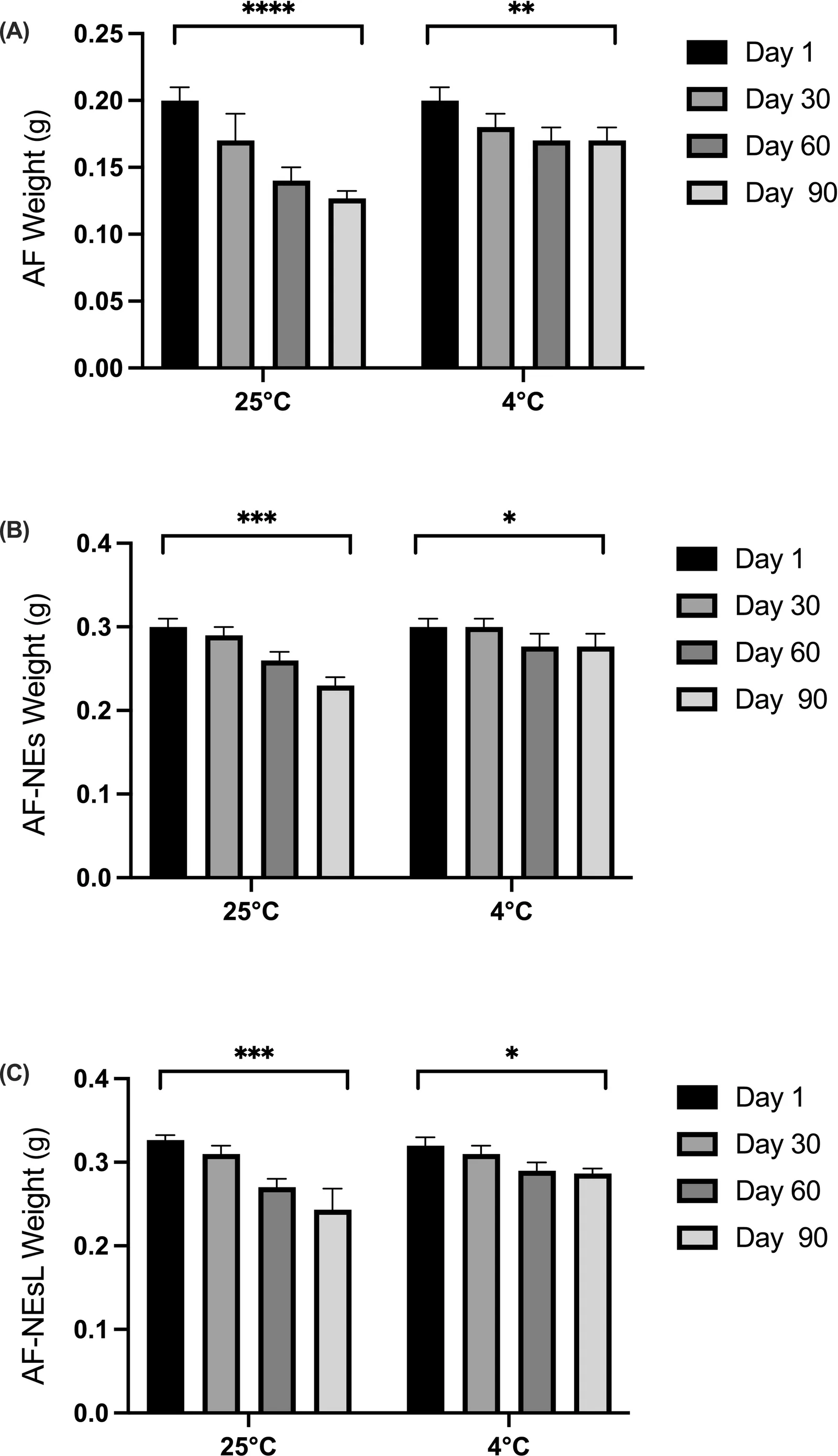
Weight stability studies of AF (A), AF-NEs (B) and AF-NEsL (C) conducted at 25°C and 4°C. Error bars: standard error of the mean. Horizontal bars with asterisks (*) indicate a significant difference between specific groups (*: p < 0.05, **: p < 0.01, ***: p < 0.001, ****: p < 0.0001) (one-way ANOVA with post-hoc Tukey test).

### 3.10 *Ex vivo* infection model

To evaluate the antimicrobial activity of selected samples (NEsL and AF-NEsL), *ex vivo* experiments were performed. In particular, the formulations and related controls (NEs and AF-NEs) were placed on 0.5 cm-diameter porcine skin discs previously infected with the reference strain *S. aureus* ATCC 6538P, as described in the Materials and Methods section. *S. aureus* ATCC 6538P is a well-characterized Gram-positive bacterium widely used as a standard reference strain for antimicrobial susceptibility testing. Originally isolated from a human lesion, this strain, although classified as Methicillin-Susceptible *S. aureus*, retains several important virulence-associated features, including a strong capacity for biofilm formation. These characteristics make it a valuable model organism for investigating pathogenic infections and microbial persistence (Wcisłek et al., 2025; Makarova et al., 2017). Interestingly, in our previous study (Maurizi et al., 2024), we demonstrated that the bacterial strains did not exhibit significant sensitivity to the NEs. However, their response profiles changed markedly following antibiotic loading. In particular, after 24 h of incubation with NEsL, MIC and MBC values comparable to those observed for free LVX were obtained, suggesting that NEsL are able to release the antibiotic in sufficient amounts within the bacterial growth medium to exert an effective antimicrobial activity, representing a model to overcome the issues related to LVX in vivo bioavailability. When the strain was put on the pig skin model, as shown in Fig. 13, after 3 h of incubation at 37 °C at sub-inhibitory concentrations, neither NEs nor NEsL were able to counteract bacterial growth. Interestingly, on the contrary, when both formulations were incorporated into the AF, a significant and a stronger reduction, with respect to the control, in bacterial viable counts was remarked.

**Fig. 13:**
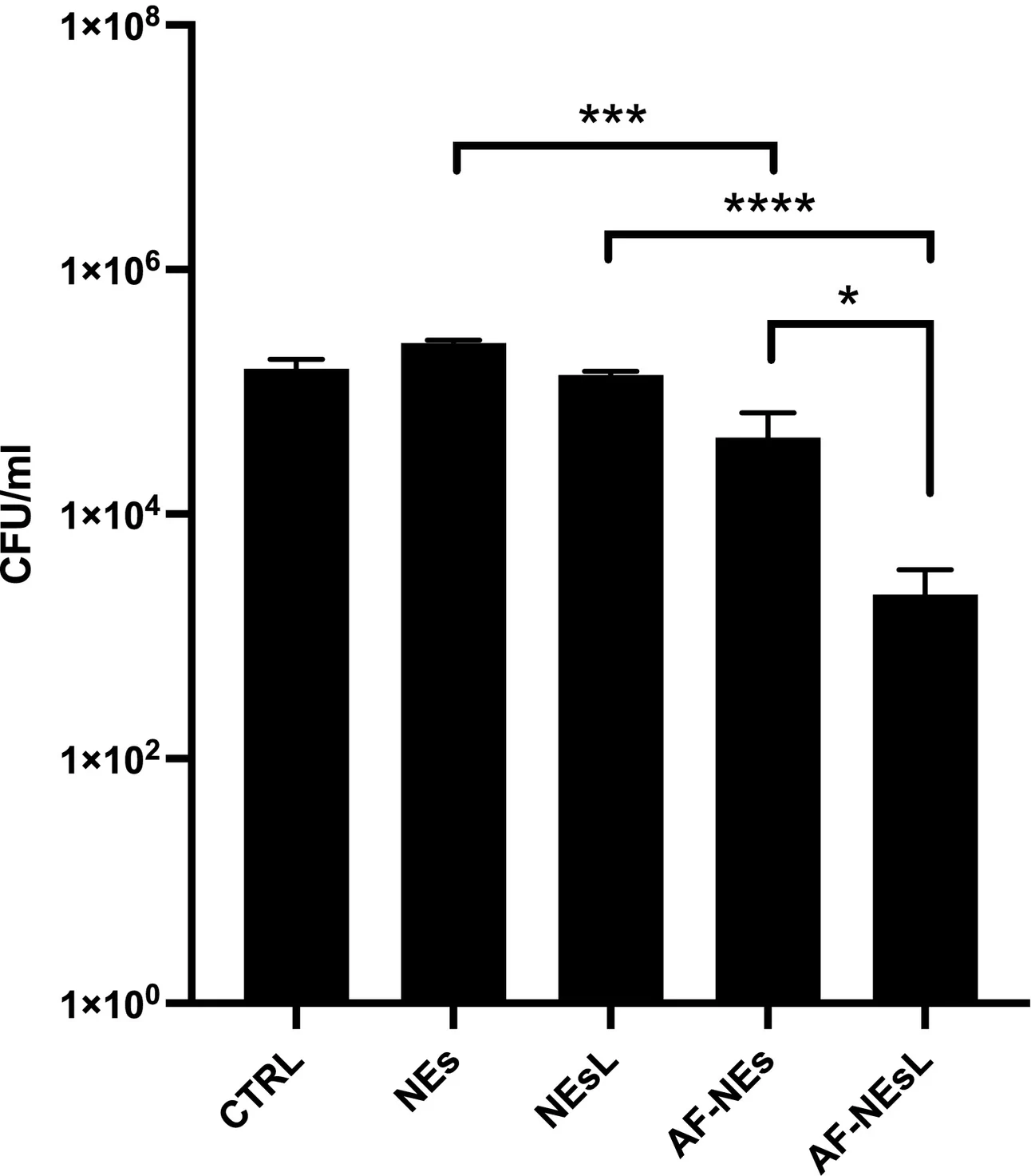
Antimicrobial activity of NEs, NEsL, AF-NEs and AF-NEsL at sub-inhibitory concentrations on porcine skin. Results are expressed as colony-forming units per milliliter (CFU/mL). Error bars: standard error of the mean. Horizontal bars with asterisks (*) indicate a significant difference between specific groups (*: p < 0.05; ***: p < 0.001; ****: p < 0.0001) (one-way ANOVA with post-hoc Tukey test).

Importantly, no synergistic or additive interaction among chitosan, lentisk oil, and LVX can be established from the present data. Nevertheless, the AF containing LVX-loaded NEs showed the most pronounced antimicrobial activity among the formulations tested, supporting further investigation of this formulation in view of its potential application as a topical treatment against *S. aureus*. The observed antimicrobial effect should therefore be considered as the overall performance of the formulation, while the potential contribution of the individual components and their possible interactions remains to be further investigated.

## 4 Perspectives and future directions

The promising results obtained from the *ex vivo* infection model support the suitability of the proposed system for topical applications and encourage further investigations to better elucidate the contribution of formulation retention and tissue interaction to its biological performance. Future and complementary studies will focus on the in vivo validation of the developed platform, with particular emphasis on further evaluating its adhesive performance under physiologically relevant conditions. The adhesive properties assessed in this study provide only a preliminary qualitative indication of formulation adherence, as the test was performed on an inert substrate and did not allow quantitative comparison among formulations. Moreover, detachment force was not evaluated. Further studies are therefore needed to quantitatively characterize adhesion strength, detachment force, and residence time on relevant biological substrates, to better assess the bioadhesive potential of the developed formulations.

The antimicrobial efficacy of the developed formulations was evaluated using the reference strain *S. aureus* ATCC 6538P, and no clinically isolated or methicillin-resistant *S. aureus* (MRSA) strains were included in the present study. The use of this well-characterized reference strain provided a standardized model for the initial assessment and comparison of the antimicrobial performance of the developed formulations. Therefore, the translational relevance of these findings to difficult-to-treat or persistent infections remains to be fully established. Validation of the platform against a broader panel of clinically relevant isolates, including MRSA strains, together with in vivo efficacy and safety studies, will be essential to establish the proposed system as a general and versatile bioadhesive platform for different therapeutic agents. Although the preparation methods for both the bioadhesive film and the nanoemulsions are simple and reproducible, their potential amenability to industrial scale-up, including the compatibility of the nanoemulsions with industrial sterilization processes, will require further investigation. Further development will be carried out on pharmaceutical optimization with the objective of advancing toward clinical translation. Once the system will be validated against additional clinically relevant bacterial strains, including multidrug-resistant isolates, future studies will also investigate its chemical and microbiological stability under appropriate storage conditions, with the aim of establishing its shelf life and confirming the maintenance of formulation integrity and microbiological quality over time.

## 5 Conclusion

The nanoemulsion drug delivery system, evaluated as a preliminary building block of the developed platform, showed suitable features for the proposed application, with no cytotoxic effects or signs of skin irritation observed under the tested conditions. In addition, the bioactive NEs, prepared using *Pistacia lentiscus* oil as a functional oily phase, exhibited measurable antioxidant activity, suggesting a potential intrinsic functional contribution to the overall characteristics of the final formulation. Based on these results, a chitosan-based semi-solid formulation incorporating LVX-loaded nanoemulsions was successfully developed and showed potential for further investigation as a topical delivery system.

The semi-solid formulations exhibited good physicochemical stability over time, with no appreciable macroscopic changes, phase separation, or significant weight variation, supporting the overall structural integrity of the system. Permeation studies using Strat-M® membrane and *ex vivo* skin models showed, for the best-performing formulations, limited trans-barrier diffusion, with a relevant fraction of the drug retained within the membrane/*ex vivo* tissue under the experimental conditions investigated. Importantly, biological evaluation using infected skin models demonstrated that incorporation of NEs or NEsL into AF was associated with reduced bacterial growth, with AF-NEsL showing the most pronounced reduction in bacterial viability among the formulations tested. This finding supports further investigation of this formulation in view of its potential application in topical delivery against bacterial infections.

## CRediT authorship contribution statement

**Jacopo Forte:** Writing – review & editing, Writing – original draft, Visualization, Validation, Software, Methodology, Investigation, Formal analysis, Data curation, Conceptualization. **Linda Maurizi:** Writing – review & editing, Writing – original draft, Visualization, Validation, Software, Methodology, Investigation, Formal analysis, Data curation, Conceptualization. **Layla Musleh:** Writing – review & editing, Writing – original draft, Visualization, Investigation. **Elvira Martino:** Writing – review & editing, Writing – original draft, Visualization, Validation, Investigation, Formal analysis, Data curation. **Chiara Tammaro:** Writing – review & editing, Writing – original draft, Investigation. **Giovanna Poce:** Writing – review & editing, Writing – original draft, Supervision, Resources, Investigation. **Isabel Schettini:** Writing – review & editing, Writing – original draft, Investigation. **Ana Isabel Fraguas Sanchez:** Writing – review & editing, Writing – original draft, Supervision, Resources, Investigation. **Xcaret Flores Hernandez:** Writing – review & editing, Writing – original draft, Investigation. **Giusi Ianiro:** Writing – review & editing, Writing – original draft, Investigation. **Antimo Cutone:** Writing – review & editing, Writing – original draft, Supervision, Resources, Investigation. **Barbara De Berardis:** Writing – review & editing, Writing – original draft, Resources, Investigation. **Federica Rinaldi:** Writing – review & editing, Writing – original draft, Validation, Supervision, Resources, Project administration, Methodology, Investigation, Data curation, Conceptualization. **Maria Carafa:** Writing – review & editing, Supervision, Resources, Project administration. **Catia Longhi:** Writing – review & editing, Writing – original draft, Validation, Supervision, Resources, Project administration, Methodology, Investigation, Data curation, Conceptualization.

## Consent for publication

All authors agree to submit this work for publication in “International Journal of Nanomedicine”.

## Declaration of competing interest

The authors declare that they have no known competing financial interests or personal relationships that could have appeared to influence the work reported in this paper.

## Data Availability

Data will be made available on request.
