# Supplementary material for: Development and characterization of a nanoemulsions-based semi-solid platform: Physicochemical properties, antioxidant and antimicrobial activities
Source: Int J Pharm X. 2026 Oct 24;12:100683. doi: 10.1016/j.ijpx.2026.100683 (PMC13742080; doi:10.1016/j.ijpx.2026.100683)
Supplement: Supplementary file 1 [file mmc1.docx]

**Supplementary Information**

**Development and Characterization of a Nanoemulsion-Based semisolid platform: Physicochemical Properties, Antioxidant and Antimicrobial Activities**

***Optimization of Advanced Formulations and NEs loaded-Advanced Formulations***

Preliminary experiments were conducted to optimize the final advanced formulation, the concentration of components and process parameters.

In particular, preliminary experiments were conducted to optimize both the composition of the formulation and the preparation process before defining the final system. The optimization strategy included the evaluation of different gelatin and chitosan concentrations to identify the most suitable in terms of film-forming ability. In addition, key preparation parameters, such as polymer dissolution conditions, stirring time, air bubble removal, were systematically investigated to ensure complete polymer solubilization, visual homogeneous layer formation. The final formulation and preparation protocol were selected based on qualitative assessment in terms of film formation, absence of cracks, voids or layer separation and reproducibility across independent preparations. The complete optimization study, including the experimental conditions evaluated and the rationale for parameter selection, is reported in table S1.

More in detail, different animal gelatin concentrations were initially evaluated for the preparation of the gelatin layer. A concentration of 1% (w/w) gelatin produced a film that was too brittle to form a continuous and stable layer (Figure S1, panel A). Conversely, at 5% (w/w) gelatin, a highly viscous solution was produced, preventing proper casting of the layer, even with increased drying time or elevated oven temperatures (Figure S1, panel B). Therefore, a gelatin concentration of 2% (w/w) was selected as the optimal condition, allowing proper processability and formation of a uniform film.

Different chitosan concentrations (1–3% w/w) were evaluated for the preparation of the chitosan layer. Chitosan concentration of 2% (w/w) was therefore selected as optimal, providing a solution with suitable viscosity for uniform spreading.

The optimization process also included the evaluation of the stirring time required for complete polymer dissolution. This parameter was optimized to ensure full solubilization of the polymer and obtain a homogeneous solution with suitable characteristics for the preparation of the adhesive layer.

Additionally, a centrifugation step was introduced in the preparation of the chitosan solution to remove air bubbles formed during magnetic stirring. In the absence of centrifugation, the resulting film exhibited irregular voids and surface defects (Figure S1, panel C). Centrifugation parameters, including speed and duration, were optimized to improve the removal of entrapped air from the polymeric solution. The optimal condition was selected based on efficient degassing while preserving the homogeneity and stability of the formulation.

Finally, the method was optimized by leaving the obtained semisolid formulation to dry at room temperature in a glass desiccator containing silica gel, which prevented layer separation, as shown in Figure S1, panel D.

**Table S1.** Complete optimization study, including the experimental conditions evaluated and the rationale for parameter selection.

| **Parameter** | **Parameter** | **Parameter** | **Parameter** |
| --- | --- | --- | --- |
| Gelatin concentration | 1–5% (w/w) | Film-forming ability | 2% (w/w) |
| Chitosan concentration | 1–3% (w/w) | Solution viscosity and homogeneous spreading | 2% (w/w) |
| Chitosan stirring time | 15 minutes, 30 minutes, 1 hour and 2 hours | Until up complete polymer dissolution | 2 hours |
| Centrifugation | Different speeds (2000, 5000 and 8000 rpm) and times (2, 5, 8, 10 minutes) | Complete air bubble removal | 8000 rpm, 10 minutes |

**Figure S1.** Evaluation of different conditions for the advanced formulation (AF). (A) AF prepared with low gelatin concentration (1% w/w), resulting in a brittle layer. (B) AF prepared with high gelatin concentration (5% w/w), resulting in a highly viscous solution preventing proper casting. (C) AF prepared without centrifugation, showing surface defects. (D) AF dried without a desiccator, showing layer separation.

***Adhesion studies***

To evaluate the potential skin-retention properties of the formulations, a sliding test was performed using a glass substrate model. Glass substrates were positioned at a 90° angle, and AF, AF-LVX, AF-NEs, and AF-NEsL were applied. The displacement of the formulations was monitored over 60 minutes at room temperature. All samples remained largely in place, indicating effective adhesion, with NEs-containing formulations showing slightly superior retention (Figure S2).

**Figure S2.** Sliding behavior of AF, AF-LVX, AF-NEs and AF-NEsL on a glass substrate after 60 minutes at 90° inclination.
